# Performance and Challenges of Service-Oriented Architecture for Wireless Sensor Networks

**DOI:** 10.3390/s17030536

**Published:** 2017-03-08

**Authors:** Remah Alshinina, Khaled Elleithy

**Affiliations:** Computer Science and Engineering Department, University of Bridgeport, 126 Park Ave, Bridgeport, CT 06604, USA; elleithy@bridgeport.edu

**Keywords:** wireless sensor network, quality of service, service-oriented architecture, Service-Oriented Middleware (SOM) architecture, data aggregation, security, heterogeneity, fault tolerance, scalability

## Abstract

Wireless Sensor Networks (WSNs) have become essential components for a variety of environmental, surveillance, military, traffic control, and healthcare applications. These applications face critical challenges such as communication, security, power consumption, data aggregation, heterogeneities of sensor hardware, and Quality of Service (QoS) issues. Service-Oriented Architecture (SOA) is a software architecture that can be integrated with WSN applications to address those challenges. The SOA middleware bridges the gap between the high-level requirements of different applications and the hardware constraints of WSNs. This survey explores state-of-the-art approaches based on SOA and Service-Oriented Middleware (SOM) architecture that provide solutions for WSN challenges. The categories of this paper are based on approaches of SOA with and without middleware for WSNs. Additionally, features of SOA and middleware architectures for WSNs are compared to achieve more robust and efficient network performance. Design issues of SOA middleware for WSNs and its characteristics are also highlighted. The paper concludes with future research directions in SOM architecture to meet all requirements of emerging application of WSNs.

## 1. Introduction

Increased use of Wireless Sensor Networks (WSNs) in numerous surveillance, healthcare, and industrial applications calls for more reliability in the transmitted data [1]. Sensor nodes associated with WSNs communicate with each other wirelessly by using different protocols and algorithms. Reliable and efficient communication between sensor nodes transmitting important data remains a major challenge in next generation WSNs [2]. Sensor nodes have several limitations such as security, data aggregation, high-level programming, localization, middleware requirements, Quality of Service (QoS), heterogeneity of the sensors’ networks, and power consumption. There is a tremendous need to integrate an innovative middleware design based on Service-Oriented Architecture (SOA) with WSNs to address the challenges associated with their reliability and efficiency.

Middleware is implemented as a web service or an interface that connects with client applications. The purpose of middleware is to control sensor data, deal with a sensor node request, and provide temporary data storage for the current sensor data [3]. Middleware controls and monitors sensor data by using intelligent mechanisms to determine when and how to query and access data that comes from sensor nodes. In some cases, the communication method between the sensor nodes needs to update and obtain a new measurement of data. The intelligent technique in middleware provides an efficient process to transmit sensor data with minimum power usage. The middleware provides a model referred to as a virtual machine with two different layers called the cluster layer and the resources management layer [4]. The cluster layer forms the cluster of sensor nodes located close to the target events. It is the responsibility of the management layer to adapt and distribute the resources required by particular applications such as security, QoS, and reliability [4].

SOA is a software design that allows communication between the hardware and applications through a secure protocol independent of the product and technologies. The communications that occur over a SOA are loosely coupled and allow for functional modifications and upgrades depending on the business needs [5]. SOA is used in a variety of industrial, military, and smart home applications. Most applications require real-time monitoring with high accuracy and reliability as applied in the European Research(EU) project called Service-Oriented Cross-layer infrastructure for Distributed smart Embedded devices (SOCRADES) for WSN systems on factory automations in industrial applications [6]. The most common applications used in smart homes are based on the concept of home energy management systems. These systems are based on one universal internet that results in a reduction of development time and cost. An example of this can be seen in the development of a peer energy cloud for monitor energy consumption through unique platforms. This platform has the capability to hide itself from the applications and allow sensors to individually measure the energy consumption for all devices [7]. Another study attempts to reduce energy in smart homes do so by using energy distributed system [8]. Similar system applied in smart homes and industrial facilities is a Distributed Operating System based on SOA (DOS-SOA) [9] and optimal control of a legacy power grid by using WSNs [10]. The SOA-DOS manages all embedded devices at a high-level of interoperability in the network [9].

The SOA is also applied in military and civil domains due to its flexibility and the interoperability of services. However, in a tactical domain like military networks, the services are mostly constrained with limited bandwidth and unreliable radio networks. This challenge is addressed by Wireless Broadband Mobile Networks (WBMNs) [11]. Moreover, SOA is integrated with WSNs for Intelligent Transportation Systems (ITS), obtaining the best results for safety and security in ITS applications. This system has modules for monitoring, management, and the user (client). This approach is deployed in parking lots which use WSNs and SOA to design suitable applications to manage this system [12].

This paper presents a systematic study of recent researches on Service-Oriented Middleware (SOM) architectures for WSNs. When compared with existing literature reviews to design an efficient system that addresses the most significant challenges, this paper makes several distinguished contributions, including security, data aggregation, message exchange, and quality of service. The paper explores various approaches based on SOA and SOM architecture to highlight possible solutions for WSN challenges. Section 2 presents background information and concepts for applying SOA middleware architectures for WSNs. Section 3 discusses the requirements of SOM architectures for WSNs. Section 4 provides the goals and challenges of middleware. Current SOM architectures approaches for WSNs are discussed in Section 5. Section 6 reviews a variety of SOA schemes for WSNs. Section 7 discusses the service composition for WSNs within SOA. Finally, Section 8 and Section 9 provide detailed analysis of comparison tables and conclude the paper with a discussion on the limitations of existing approaches.

## 2. Background and Concept

### 2.1. Middleware Architectures for WSNs

**Figure 1:**
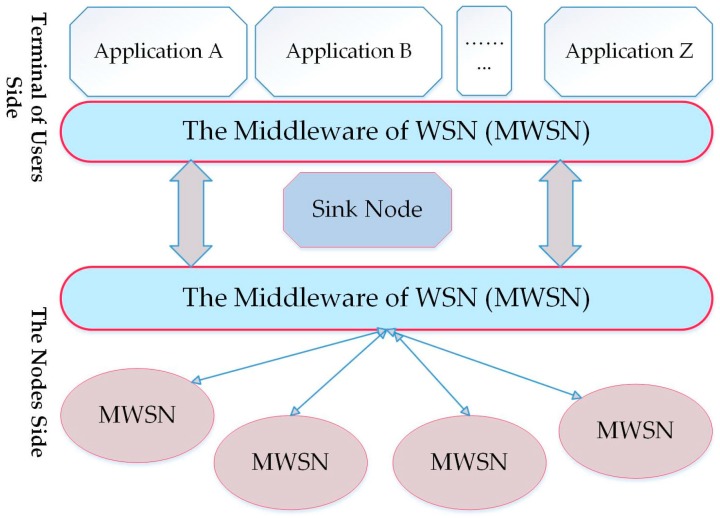
Middleware Architecture for WSN [13].

**Figure 2:**
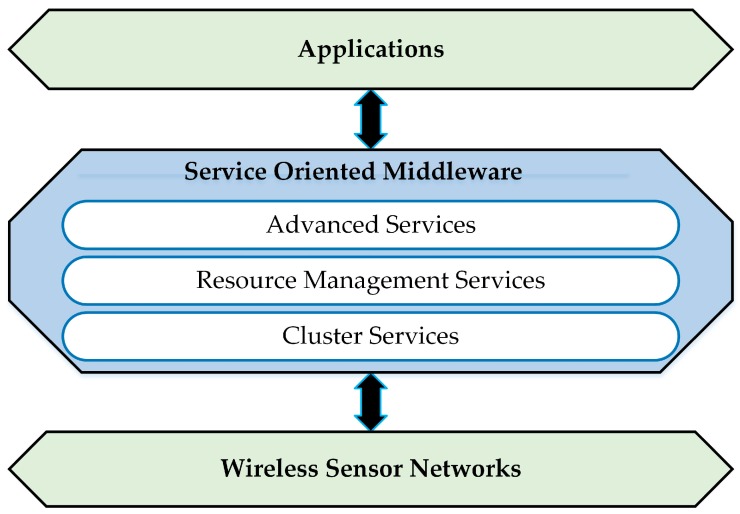
The SOM Architecture Layers for WSNs [4].

The middleware architectures for WSNs have the ability to distribute sensor nodes, sink nodes, and high level applications [13], as shown in Figure 1. This middleware does not support SOM architecture that is integrated with WSNs [4]. SOM architecture is a designed middleware similar to WSN middleware with a new layer called the advanced services layer [4]. The architecture consists of three layers, which are the cluster services layer, resource management services layer, and advanced services layer. The advanced services layer provides services for security, QoS, and reliability applications [4]. Each layer provides services for the above layers as shown in Figure 2.

Middleware provides many advantages when applied to WSN applications. These advantages range from hiding the complexity of the network communication, dealing with the heterogeneity of applications or devices, and managing system resources. The components of the middleware architectures are used to integrate WSNs with user applications while the complexity and heterogeneities of the hardware and software are hidden [14].

**Figure 3:**
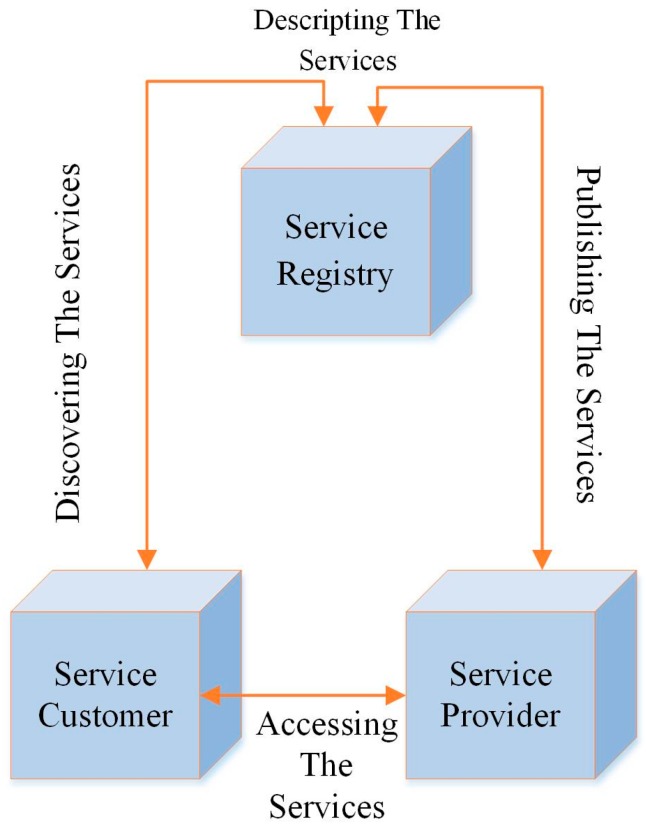
Service-Oriented Architecture (SOA) [15].

The literature discusses a new and emerging architecture called SOA, where each component acts as a service. It enables the software services to interact with each other to execute and complete numerous tasks. The SOA services communicate through different standard languages such as Extensible Markup Language (XML) and Simple Object Access Protocol (SOAP). The challenges mentioned above can be addressed using the SOM architecture. The SOA can be applied with or without middleware that allows different applications to interact with various networks. SOA is a framework design that enables various applications to be developed by using loose coupling and interoperable services. The SOA consists of different components including the service provider, service registry, service customer, and message-based interaction protocol as shown in Figure 3 [15]. Moreover, SOA enables different services for Heterogeneous Cyber-Physical-Systems that can be selected and shared among various applications as proposed in [16].

### 2.2. Service-Oriented Middleware (SOM) Architectures for WSNs

The Service-Oriented Middleware (SOM) architectures are used to make service available and easily accessible by using standardized protocols without any concern about the details of implementation. SOM architecture helps WSN applications to develop over traditional development platforms which address these challenges. The WSNs connect to the SOA through different elements such as the middleware. In [17], SOM architecture considers the WSN as the service provider for user applications [17]. The middleware is implemented in or out of SOA, which is important for integrating/exchanging messages. Broker Registry is also responsible for allowing service discovery and making communication easier in SOA. Middleware has the reliability of messaging and guaranteeing that the messages reach the receivers. It has the ability to store messages for a long time and send multiple messages in parallel, resulting in increased speed in the execution of data messaging.

The heterogeneous nodes in WSNs can impact the entire network’s capability. In the case of a mismatch in data formats and structure exchange between nodes, the system should provide a mechanism for heterogeneous nodes to handle mismatch data, since all nodes communicate only with nodes of a similar data structure and exchange data formats model. The mismatching of communication types exists due to the implantation of different formats of data. There are some techniques that separate the service form application, i.e., dynamic allocation of resources and function level, which allow different applications access to similar nodes. This causes limitation, which can increase the complexity of the middleware’s developed code. Most data aggregation techniques within SOA deal with simple data such as temperature, humidity and others. In this case, it is difficult to deal with complex data such as images and videos. The distributed middleware is used to combine services via networks. Logically, the network is located in the network layer but physically exists in the nodes [18]. The Service-Oriented Software Architecture is based on an adaptive middleware that is used for sensor networks. These nodes are connected only by the services of the middleware [19].

## 3. The Requirements of an SOA for WSNs

The requirements of a Service-Oriented Architecture (SOA) lay in the fact that the components of an application provide services to other components. In order for this to take place, communication is done over a network. Many different applications and their components on the same network can effectively cooperate with each other on the basis of SOA. SOA provides a platform where diverse services can exchange information over the network without human interaction or changes to the program [17].

The challenges of SOA are diverse. They can range from management, to testing, to security issues. It is very common for applications within a system with SOA to generate thousands of messages to be transmitted across the network in many different directions. The management of these messages coming from different applications could be a huge challenge. In a more complex SOA system where third party companies and outsourced systems are connected to the same network, management of those messages can be even more complicated. Security in SOA is challenging because it should be provided at appropriate levels within the application. It is almost impossible to provide security for the services that can be used by other applications. In a conventional SOA architecture, testing capabilities can be a big challenge. Providing distinguished tools for testing in the SOA space can be a complicated task. If accomplished, the architecture would have many flaws, which would be difficult to rectify within the application [17,20].

One requirement of the middleware is to provide low power communications while making efficient use of memory and the transmissions. The components of the device should be set in an efficient way where sensing and data processing over the network flow well. Depending on the needs of the application, the components should be turned off to save energy while providing maximum efficiency [17]. In a middleware architectures for WSNs, one of the challenges is to provide scalability and maintain topology of the network. The network topology changes based on malfunctions in the device or one of the interfaces. In such event, it is difficult to provide an error-free network that can accommodate such obstacles. The heterogeneity of the model is very challenging because it is continuously trying to find a common ground between the hardware and software applications. Effective interfacing of the two can be complicated and often prone to malfunctioning. It is very difficult to manage networks since many applications are running for a long time. In addition, the messaging and communication between the applications can be too complex for the network to handle and manage. The design principle of application knowledge is another significant challenge because the tuning and mapping of the network in correlation to its applications are highly essential [17]. The Quality of Service (QoS) includes accessibility, reliability, robustness, timeliness and the optimum security of WSNs. The QoS should be very high because of the unique nature of WSNs and the data transfer required for an effective communication [21].

## 4. The Goals and Challenges of Middleware Architectures for WSNs

Middleware architectures for WSNs have various challenges as discussed below [22,23].

### 4.1. Scalability

Middleware architectures should be scalable to dynamic resources and interfaces to ensure superior performance as the size of the network grows. Scalability is challenged when any change occurs on large-scale networks. For example, when adding new nodes, the network should adopt and synchronize them with the existing nodes. An efficient middleware design is capable of maintaining a large network and adapting to any changes that occur without impacting network performance.

### 4.2. Heterogeneity

The heterogeneity among the hardware, communication devices and configurational operations have to be granted for the middleware. The heterogeneity of the components may be an issue in large-scale applications of wireless sensor networks.

### 4.3. Data Aggregation

In order to minimize the volume of data for transmission, a sensor network uses data aggregation quality. This ensures that redundant data is not generated in the memory, saving costs through memory usage and energy through processing time. This is a more data-centric approach in comparison to the conventional, address-centric approaches.

### 4.4. Managing Limited Battery Power

With smaller, more compact sensors, the available battery power is always limited. The systems are designed to manage limited power by designing efficient processes and capabilities of the sensors. Mechanisms to ensure efficient power consumption are necessary for advanced wireless sensor networks.

### 4.5. Quality of Service (QoS)

It is important for the wireless networks to support QoS as it pertains to the accuracy of data, coverage and tolerance. The quality of service is important on the application level as well as on the network level. The QoS considers the resource constraints in new and adaptive WSN designs.

Providing most efficient and suitable nodes to the client who is in need of the resources has been a major problem in cloud computing. The ability of the system to efficiently locate and provide the needed resources to the clients is vital. Recently, some researchers [24,25] have tried to increase and optimize the QoS by using computing environments such as Cloud/Grid systems that comprise of several trusted nodes to manage local resources individually. A trust model is associated with each node that accurately evaluates the trustworthiness of its communicating clients [24]. The time-consuming and inefficient process of exploring the whole node space is avoided by allowing each node to efficient allocating resources by finding suitable collaborations. The authors showed the employment of a decentralized approach using Hypertrust where the nodes are organized in an overlay network given the criteria by the client. The Hypertrust gives the client an efficient way of searching for available resources while empowering the nodes to use their respective trust models to limit the search. The unique node called Task Allocator (TA) allows clients to delegate the selection processes of the task as well as improving the overall QoS.

Another approach, called the partnership based approach [25], is introduced to maximize the QoS by improving and optimizing the global QoS for the large-scale federated resources [25]. This approach combines the trust models for software agents to support the federated computing nodes. The intelligent agents support the model computational nodes which can manage the Friendship and a Group of Membership (FGM). The Friendship and Group Formation (FGF) algorithms used in this approach enable the federated nodes to select their FGM that can increase and improve the global QoS. The authors in [25] showed metrics that allow most suitable resources in such Grid/Cloud systems. Potential collaborations and competition between resources providers for clients’ needs are explored by the federation of computing.

### 4.6. Security

With popularity and advancements in WSNs, large chunks of sensitive information are sent over wireless networks. This information can be easily hacked by malicious intrusions and internet attacks. The integration of security parameters in the system’s design is necessary to achieve protection.

Most of the middleware focuses on resource distribution, management, and the communication efficiency of the sensor network. However, data aggregation mechanisms, security methods, and resource distribution still remain massive challenges. Security must be part of the middleware design for approaches that use multiple networks’ distribution. The middleware reduces the probability of errors or failure by managing multithreads efficiently. Different security mechanisms should be increased by developers of networks during the design of middleware based on SOA. The abstraction layer, wrapping mechanism, and intelligent interfaces are used to address issues of heterogeneous data fusion. The security solutions are considered in several SOM architectures approaches. [26] Proposes a generic security service for SOM architecture frameworks that provides various independent security services such as authorization, authentication, and access control.

The SOA based on middleware is designed for Security and Surveillance WSNs with Commercial Off-The-Shelf (COTS) used to program and deploy the data processing applications after analyzing a web service [27]. This approach provides a unique, distributed data processing application in WSNs for Mobile Ad-hoc and Sensor Systems (MASSs). The architecture provides support to complex monitor applications aimed at global security, loose coupling, auto-organization mechanism, simplified connection heterogeneity, and interoperability [27].

In addition, the security mechanisms can be achieved by end-to-end security auditing for SOA as introduced in [28]. This solution provides two new components called Taint Analysis (TA) and Trust Broker (TB) with some advanced features that take from WS-Security and WS-Trust Standards [28]. TA monitors the interactions of services at runtime and checks information flow between them, which can detect particular events. TB is considered a trusted third party responsible for maintaining end-to-end auditing in the information flow into client requests [28]. In this architecture, the service providers should register themselves closed to TB, which allows user verification by the security of the service providers via TB.

### 4.7. Fault Tolerance

Many studies are focused on how to recover the system from failure. SOAs have an important feature that can maximize information reuse by separating the implementation of services from the interfaces and enabling failure-resistant networks. The Service-Oriented self-healing approach referred to as “clinic” is proposed in [29]. The self-healing service can, with help of SOA, detect faults and heal them, isolating them by only using information that is available from other services in different networks. The evaluation of the self-healing approach is applied on communication faults through a routing protocol called Multi-path, Multi-hop Hierarchical Routing (MuMHR) [30].

## 5. The Taxonomy of Middleware Architectures for WSNs

**Figure 4:**
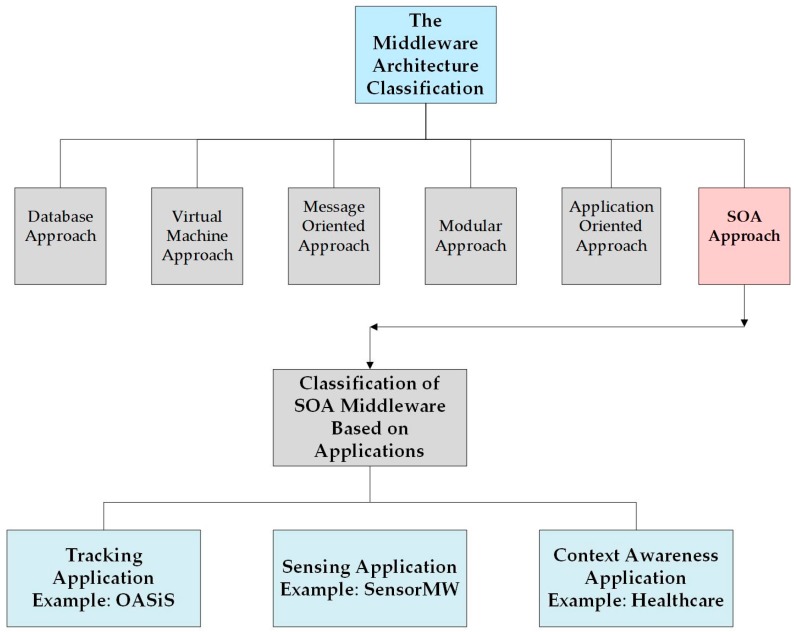
The Classification of Middleware Architectures for WSNs.

The middleware architectures for WSNs have been used widely to reduce the complexity of WSN applications. The classification of middleware architectures approaches are proposed in the literature [22,31]. The middleware architectures based on SOA for WSNs can be classified based on the applications targeted as shown in Figure 4. Additionally, Table 1 presents the comparison between different middleware architectures designed for WSNs.

### 5.1. Database Approach

This approach considers the entire sensor network as a distributed database. The limitations of this approach is that it does not support real-time applications and only provides approximate results. The example for this middleware architecture is Sensor Information Networking Architecture (SINA) [32]. The SINA is capable of monitoring changes within the network.

### 5.2. Virtual Machine (VM) Approach

The Virtual Machine (VM) middleware architecture is a flexible approach that allows the developers to write the applications in separates modules. The modules are distributed in a network by using specific algorithms. Even though the issues related to the utilization of the resources and power consumption are addressed in this approach, the limitation of the VM approach is the overhead.

### 5.3. Message-Oriented Approach

This middleware approach is used the publish/subscribe mechanisms which can facilitate the message exchange between the base station and the sensors nodes. The advantages of this middleware is that it supports loose coupling and asynchronous communications between the sender and the receiver.

### 5.4. Modular Approach

This approach divides the applications as modular programs that help the integration and the distribution through network by using mobile codes. The limitations of this approach is that it does not support the heterogeneity sensors hardware.

### 5.5. Application Driven Approach

This middleware allows the application to identify their QoS requirements then can modify the network according to application needs. The Middleware Linking Application and Network (MiLAN) is one of the examples of the application driven [33]. The limitation of this middleware is not supported the heterogeneity sensors hardware.

### 5.6. Service-Oriented Architecture Approach

The middleware based on SOA is proposed in detailed in Section 5. The Service-Oriented Middleware (SOM) architectures are presented below and is classified based on the applications targeted.

#### 5.6.1. The Sensing Applications

SensorsMW is a SOM architecture that allows applications to configure and adapt to the low-level hardware based on their particular requirements. SensorsMW has been developed for vent monitoring and periodic measurements. This middleware is used to test temperature measurement applications.

#### 5.6.2. The Tracking Applications

The OASiS is a tracking application for example fire detection and vehicle tracking [34,35]. The WSN-SOA has been tested for surveillance applications with the ability to detect seismic vibrations [36,37].

#### 5.6.3. Context Awareness Applications

The middleware has been designed for context awareness applications and testing for healthcare and smart environments [38,39,40,41].

## 6. Service-Oriented Middleware (SOM) Architectures Approaches for WSNs

The SOM architecture is the best platform to develop WSN applications to address hardware challenges such as QoS, security, and heterogeneity. The following is a brief description and summary of the selected approaches that are considered SOM architecture for WSNs.

An open sensor middleware model based on the SOA for WSNs should have the ability to integrate, in real time, context data with flexibility, reusability, programming abstraction, and simplicity. In addition, many studies consider the network-embedded devices in different applications, such as managing enterprise architecture [42], smart home and industrial applications. These applications can be classified into two categories: SOA-ready devices and SOA not-ready devices [43]. The issue of integrating WSNs into IP-based networks and Internet is addressed in [43]. It provides solutions for implementing SOA based on SOA not-ready devices. A micro SOA model is implemented based on µIP protocols that only use Hyper Text Transfer Protocol (HTTP) philosophy instead of HTTP protocols [43]. The exchanged data can be between network devices on the same layer or between the embedded and middleware layers through efficient lightweight protocol called JavaScript Object Notation (JSON) (instead of XML format) [43]. JSON can reduce overhead and power consumption, request size, and complete request time. The µSOA uses the middleware layer. The middleware layer manages access to WSNs by filtering and protecting the system. The filter mechanism removes unnecessary information from any HTTP request. Other mechanisms the middleware provides are security, domain name services, and authorization. However, this middleware does not address the issue of a heterogeneous network [43]. Similarly, the middleware can be designed based on a function block programming abstraction for a WSN that enables the operations to be done in a dynamic environment to reduce overhead and complexity. These features are completed by applying SOA with a Mobile Agent (MA) [44].

### 6.1. USEME

**Figure 5:**
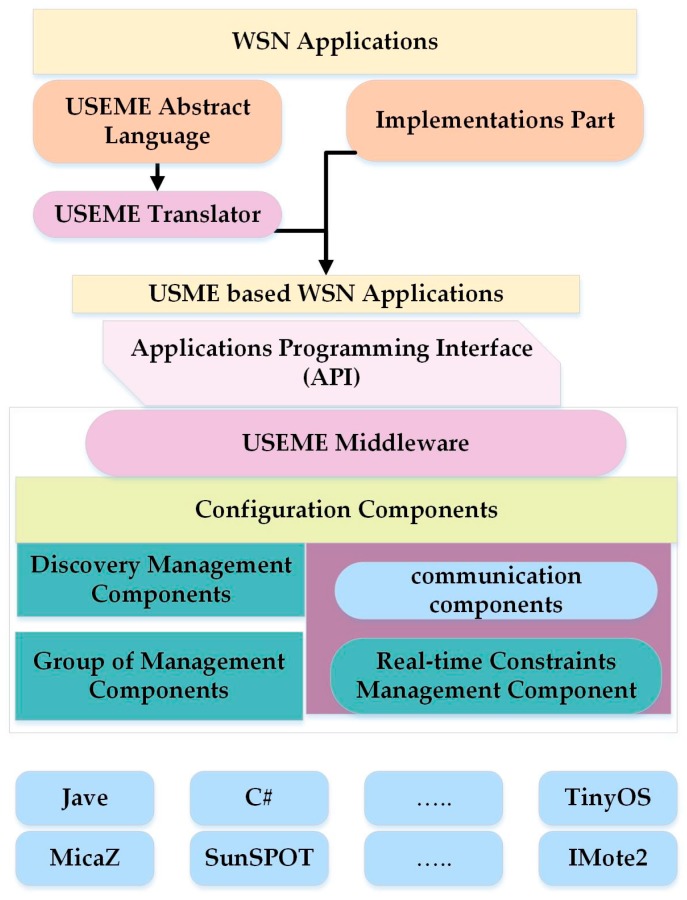
USEME Architecture [45].

In [45], the authors propose Ubiquitous SErvices on Mote (sensor) Environments (USEME), a new framework that uses Service-Oriented high-level programming models [45]. It also supports middleware development of Wireless Sensor and Actor Network (WSAN) applications [45]. Efficiency and scalability are realized through the middleware, which has various sensor nodes that can share a mutual behavior and control the use of services. The drawbacks of priority and deadline are considered in this approach, which can deal with the real-time actions of the services requirements. This approach combines macro-programming with node-centric programming. Different prototypes are developed by using three motes: Crossbow family MicaZ motes, Imote2 (Crossbow Technology, Inc., Milpitas, CA, USA), and SunSPOT, as shown in Figure 5.

The authors of [45] did not provide data on whether the architecture is a distributed or centralized model, or on the methods of used services. The proposed framework did not consider the accuracy and QoS constraints. The solution for this limitation is to provide an application designed to define a set of services, nodes, and events. This approach should be supported in real-time, which can allow the programmers to recognize (define) QoS among the services by using communication. The study in [46] uses the same techniques as above but focuses on middleware to support USEME. This Service-Oriented Framework is used to deploy lightweight services on the sensors and actors. Two different prototypes are used to implement this approach, which are SunSPOT devices and Imote2.Net from Crossbow. The middleware provides an easy way to address any differences in the nodes as they pertain to the dynamic and logical relationship between the services in the application. The features of this middleware make the network more secure, facilitate updates, and ensure controlled deployment.

### 6.2. SOMDM

In [47], the authors proposed a unique, SOM architecture with a Message-Driven architecture for an ambient aware sensor networks (SOMDM) technique [47]. The limitations of web service as well as time, power, and memory consumption issues in the physical layer are addressed in this middleware. This approach has enabled the SOA to reduce process load in real-time during query processes, warning the system, and performing processes for ambient aware sensor networks. The system approach uses the data filtering mechanism which has been used to filter the event of interest. The object codes are the nodes in a sensor network that will follow the ambient program model, which permits nodes to communicate in two asynchronous ways. The object codes should go to a data filter box with intelligent mechanisms to filter normal and abnormal data. Moreover, normal data goes to the Management System Database (MSDB), which stores the data that comes from the DataFilter Box and can be used to query other parameters. This approach is tied to abnormal data, which should go to the message queue through a Java Message Server (JMS). Then, it Normalizes the Message Router (NMR) using a fast response time in warning messages. The NMR can reduce the load of discovering and subscribing the route. It provides the best solution for communication time between services. This approach does not consider security mechanisms for internal and external communication between the nodes and client. The quality of service should be considered in this approach in order to obtain better accuracy and faster operations.

### 6.3. Mobile Web Services

In [48], a Mobile Web Service (Mob-WS) middleware that provides the best management and representation of wireless networks was designed. The Mob-WS is used as a back-end resource for in-network computations. The Mob-WS middleware addresses the issue of inflexible collector nodes. The middleware deployed with hosting a long-lived asynchronous services. The Mob-WS middleware is deployed on the collector node, which can make it independent of any transmit protocols. The collector node concept is used to perform Mob-WS base in-network that can cooperate, control, and monitor. It is the best representation of the network. The service processing model is based on in-network services, and these services are implemented on the sensor by using the computation in wireless networks [48]. This method increases the scalability of the network and makes decisions locally based on the sensing data [48]. The limitations of Mob-WS designs do not provide mechanisms to secure accessing to the services or managing the QoS on the Mob-WS. It cannot handle multi-interfaces.

### 6.4. MiSense

In [42], the authors proposed MiSense, Service-Oriented, components-based middleware layers that support the distributed sensor applications with a different performance of requirements [42]. The MiSense middleware provides an abstraction layer in between an underlying network infrastructure and the application. In addition, it provides an abstract programming model to the WSN application that can maintain the balance between network lifetime and QoS requirements for the application. The content-based, publish/subscribe service, provided by MiSense, enables the designer of any application to adapt to the services. MiSense also helps break down the middleware into different layers. The layers can be self-contained, and interact with the components that address the issues of tension between the requirements’ optimization, flexibility, and the ability to develop reusable WSN applications with efficient energy.

**Figure 6:**
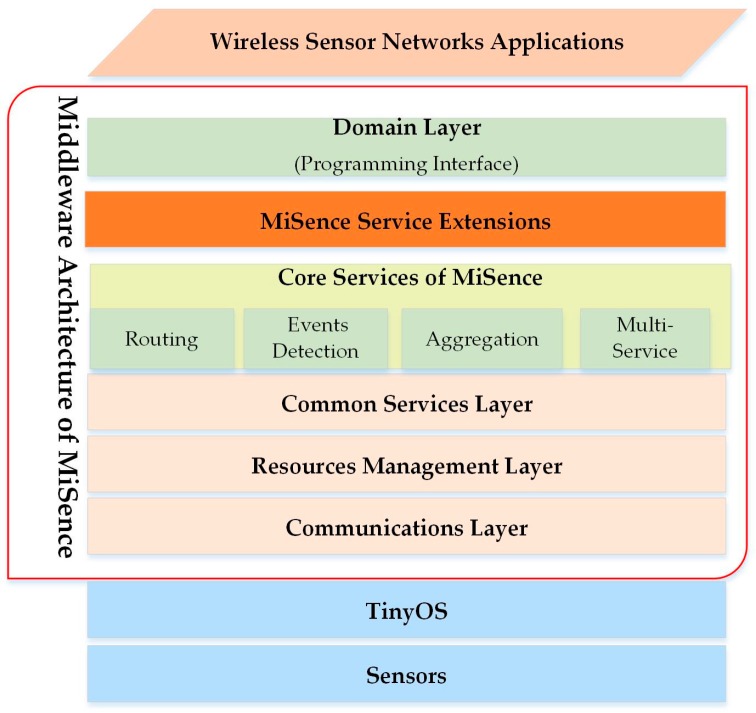
MiSense Architecture [42].

The middleware has three layers: the communication layer, common service layer, and domain layer, as shown in Figure 6. They handle data aggregation, event detection, routing, and topology management. This approach uses adapted rules for the middleware, which can increase the data accuracy and bandwidth. The energy consumption decreases by an increased data rate and changes some sensors into the sleep state mode [42]. The MiSense does not support heterogeneous data that comes from different networks. It is also dependent on TinyOS (TinyOS Alliance). This approach does not determine the standard of SOA used between the gateway and the applications [42]. This SOA has flexibility and interoperability limitation between the various platforms provided in this approach. Since binary forms are used for remote procedures, the execution of SOA applications can be slow. The results can increase the processing time and energy consumption.

### 6.5. Sensors MiddleWare (SensorsMW)

In [49], the SOM architecture is used for QoS configuration and the management of the WSNs. The authors presented Service-Oriented, adaptable, and flexible middleware (SensorsMW). This middleware supports the dynamic management of heterogeneous data. The middleware has the capability to hide the complexity of low-level sensor devices [49]. Once the SensorsMW abstracts the WSNs, it acts as a gathering service and easily integrates into the enterprise information system. The applications collect the sensed information by using a web service. Consequently, the SensorsMW allows high-level applications to configure a data collection level for the WSNs in a simple manner. This approach enables the application to collect data by using a web service, which can guarantee flexibility in the delivery of the data. Furthermore, this architecture enables applications to independently negotiate from run time by using a technique called the contract negotiation approach, based on a Service Level Agreement (SLA) [49]. SLA stops the application from requiring knowledge of the other QoS applications. The SLA enables the application to reconfigure and maintain the network within its lifetime. Every end-device node contains Crossbow MicaZ (Crossbow Technology, Inc., Milpitas, CA, USA) [49]. Every node has TinyOS 2.0 (TinyOS Alliance) [49]. The implementation only focuses on service level management and does not provide any mechanism to handle a secure execution or communication. Typically, in WSNs, a faulty node is factored into the performance of the system in order to generate the correct execution. Unfortunately, this approach does not take this fact into consideration. In addition, the resource management of the system does not support any node with low capacity. The details of QoS parameters, resource surveillance, scalability, and data evaluation are not provided.

### 6.6. OASiS

The OASiS is an Object-centric, Ambient aware Service-Oriented Sensor network applications, and Service-Oriented Framework introduced in [34]. The OASiS middleware includes various services, such as a dynamic service configurator, node manager, and object manager [34]. It can easily provide dynamic service discovery and configuration, data aggregation, and support heterogeneity (the application developers aren’t required to have any experience in sensor programming). The middleware architecture is supporting OASiS and is capable of tracking the application. The ambient aware sensor network consists of efficient mechanisms that can detect failure if any node drops out during the application execution or communication. The network application is retrieved by applying an isolation and recovery technique [34], providing a stable configuration achieved by taking some advantages of OASiS-SOA [34].

The authors introduced the sensor network application in [35] that is obtained as graphs of modular and autonomous services with determined interfaces which allow them to be published, discovered, and provide a mechanism to integrate the services from a heterogeneous sensor system [35]. The SOA model allows the composition of a dataflow application [35].

### 6.7. QoS for SOM Architecture

The Quality of Service (QoS) can be improved by applying strategies of dynamic service selection. These strategies are used to achieve a high level of QoS for WSNs and the lifetime of the network can be increased in this simple manner [50]. The Service Node Selection Algorithm (SNSA) locates the most efficient and effective service nodes to take part in composite function. In this case, the SNSA guarantees a minimum level of quality by choosing services that meet quality and network routing requirements. This mechanism enables the operation to execute with minimum time and power consumption [50].

### 6.8. SOMM

**Figure 7:**
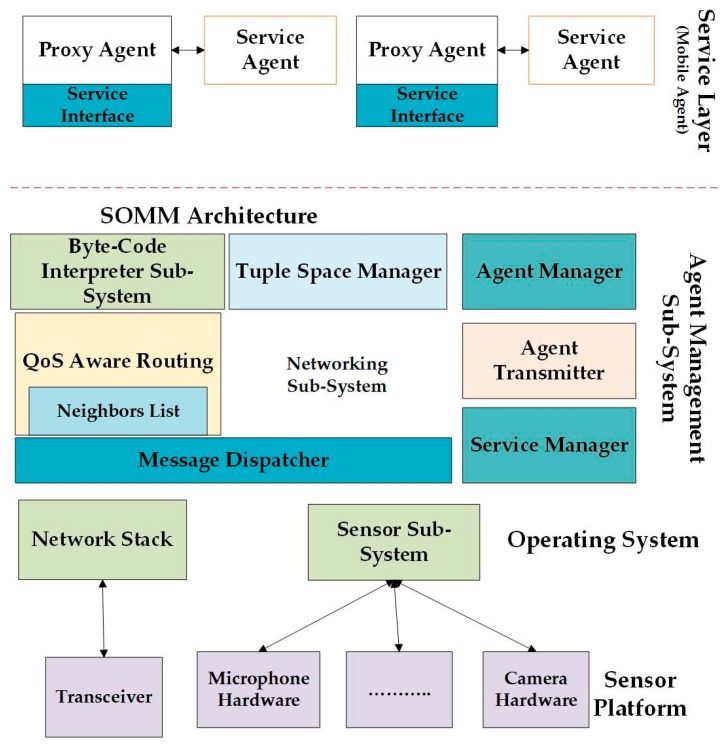
SOMM Architecture in the Server Node [51].

The Service-Oriented Middleware (SOM) architecture called (SOMM) is described in [51]. It can support the application development for Wireless Multimedia Sensor Networks (WMSNs) [51]. Several middleware designs are proposed for WSNs but this middleware is not suitable due to its constrained resources. SOMM consists of two components that are service registry servers [51]. SOA is used in SOMM, which leads to scalable and dynamic server node networks which can provide several services to different clients [51]. In this case, the network has the ability to handle many clients simultaneously and add new functions to the network [51]. The application code size is decreased by using a Virtual Machine (VM) as middleware, which supports the reprogramming of the nodes. The VM is located between the application layer and the operating system. The VM provides code mobility that is helpful for Generic WMSN (GWMSN). The overview of the middleware solution [51] is shown in Figure 7. The codes of each service are stored in specific nodes that have enough memory space (repository) to act as the mobile agents [51].

SOMM only supports Transmission Control Protocol (TCP) binding, which is in binary format, not SOAP. HTTP binding provides an overhead and increases the power consumption of nodes. The transmission of multimedia in WMSNs is supported by using some of the middleware advantages, heterogeneous nodes, and QoS. The cost of the application development is decreased while improving the scalability and modifiability of the network, which can increase power efficiency [51].

Additionally, the authors in [52] introduced a Service-Oriented Agent-based Middleware called SAWM based on a network architecture that is proper for WMSNs [52]. The middleware of WMSNs handles QoS, managing bandwidth network heterogeneity.

### 6.9. TinySOA

TinySOA enables programmer access to WSNs from an application by using Service-Oriented API [53]. This approach helps integrate a WSN with the internet application, providing an abstraction for the developers’ applications. The TinySOA acts as a basis for the middleware system and has the ability to allow application developers (that do not deal with low-level of WSNs) to obtain data from the sensors. The middleware helps integrate all the elements into the architecture.

**Figure 8:**
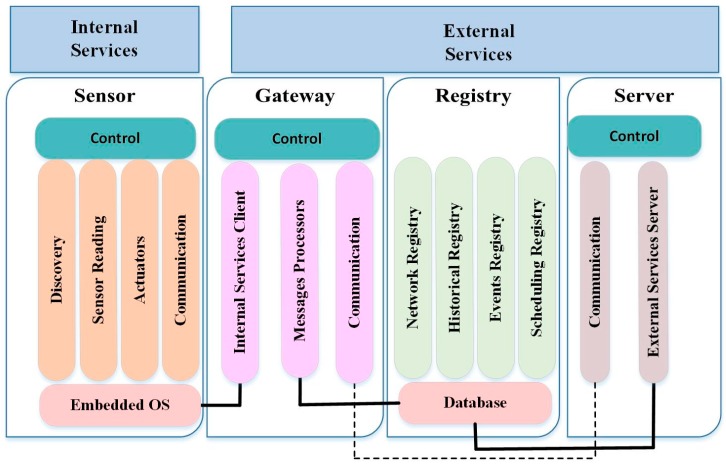
TinySOA Approach [53].

TinySOA consists of two types of services: internal and external, as shown in Figure 8. They are provided by the node, gateway, server, and register components. The mechanism of TinySOA provides node discovery and gateway for the WSN infrastructure. The gateway component is a bridge between external applications and the WSN. The hardware platform of TinySOA includes MicaZ motes (Crossbow Technology, Inc., Milpitas, CA, USA) [53].

### 6.10. ESOA

**Figure 9:**
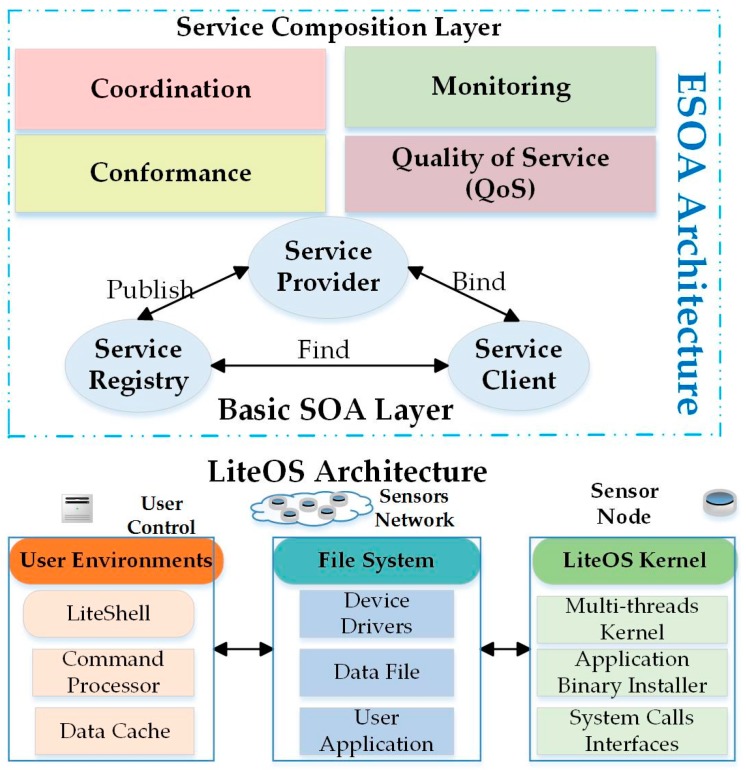
ESOA Architecture [54].

Another solution to the problems generated by an SOM architecture approach is the Extended Service-Oriented Middleware Architecture (ESOA). The ESOA, as discussed in [54], provides integrated services, customizes sensor networks, and manages applications. The ESOA is inserted above the actual SOA model and below the LiteOS operating system, as shown in Figure 9. This architecture allows users to develop new applications through mix-and-match services without any programming efforts by the developers. Since this system supports the heterogeneous WSNs, it executes various applications on multi-platforms. The ESOA approach is limited because it does not provide any methods of user accessibility data collection to the services. Also, ESOA is not applied in real time.

### 6.11. HealthCare Approaches

Within the healthcare industry, SOA is widely used to improve the transmission of important patient information. By linking the data to the healthcare community, doctors and caregivers have remote access to all of their patients’ daily activities.

**Figure 10:**
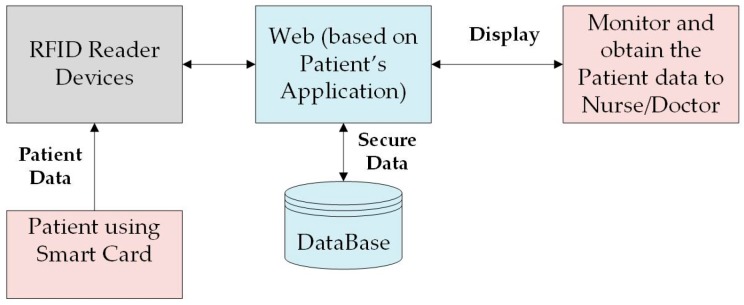
SOA-based Patient Monitoring System [41].

The monitoring system for a patient using SOA as shown in Figure 10 [41], An SOA approach is applied into WSNs to design different applications to monitor the patients for long periods of time [20]. Through SOA, the sharing of patient data has become cost-effective and secure. In [41], WSNs are introduced as an integrated with a web service, using context-aware SOM architecture that increases system flexibility. A web service combined with Radio Frequency Identification (RFID) is necessary to manage patient information. It is responsible for collecting, storing, and making clinical data available [41]. The context-aware service searches the patient information and obtains the most accurate output without errors. In its own capacity, RFID can access secured patient information. RFID is designed as a smart card accompanied with a verifiable, individual patient photo ID to obtain patient history that helps doctors give accurate diagnoses with less fault detection. This process produces an improved QoS and reduces costs.

### 6.12. Other SOM Architectures for WSNs

The implementation of SOM architecture is based on Devices Profile for Web Services (DPWS) architecture that contains new layers [14]. The SOM architecture provides a mechanism that mediates data exchange between a web service and the heterogeneous sensors [14]. The limitation of resource constraints in WSNs are addressed by using optimization mechanisms that can reduce the overhead required through using traditional WS. The energy-aware mechanism is important for extending the network lifetime. This architecture focuses on sensor nodes that impose restrictions on the resources and data aggregation. Also, SOA controls the energy consumption of each sensor by reducing transmission messages to the base station using multi-hop communication. DPWS used inside the middleware has various new components that include binary encoding, WS-eventing, and a roaming manager. The binary encoding mechanism is used instead of an XML message to reduce the overhead generated by XML. Before messages are transmitted between the layers, they should be encoded in a binary format. WS-eventing removes the requirement for necessary periodic call services and the user can subscribe to the interface of service eventing [14]. Also, WS-eventing has the ability to report to clients that a change in the data occurred. This method helps save the limited network bandwidth [14]. This approach lacks the mechanisms that can handle interaction with different components.

Another SOM architecture approach to consider is called the Service-Oriented Wireless Platform for Acquisition and Control (SOWPAC) [55]. SOWPAC is introduced in [55] as a design with an open interface to have an efficient and cost-effective deployment. Most of the platform studies focus on the industrial acquisition and control of using WSNs, which are considered only at the network, node, or data abstraction level. This consideration lacks a holistic point of view, which can limit the use of these approaches [55]. The middleware API is used to manage data, facilitate communications, and define the processes of data exchanged between functional blocks. The SOWPAC consists of a basic element called Remote Terminal Unis (RTU), which is responsible for remote sensing and actuation. The WSN-gateway is used as an intermediate element to send data from the RTU to the Central Control Point (CCP) through the WSN. The internal database in an RTU [55] can store sensing data and has the capability to recover from any failure of communication and reset the entire network. The Central Control Point (CCP) provides a user interface and application programming to manage platforms, data, and services. It also offers a Service-Oriented Protocol based on SensorML that provides an easy way to integrate a web service with high-level applications. The WSN-gateway is responsible for translating data and meta-data [55].

In addition to an Open Framework Middleware (OFM), [56] introduced a comprehensive framework designed a middleware architecture for WSNs. OFM architecture consists of a protocol stack which has some limitations, such as overhead and load on execution. The Hybrid Native Architecture (HNA) [56] addresses the drawbacks of the OFM by removing the stack-based protocol layers. It runs the Service-Oriented OFM Micro-Middleware through the device abstraction level [56]. The solution of HNA lies within system distribution services and the management of node operations which can interact with low level resources. In order to solve the above-mentioned issues, HNA should collaborate with OFM functionality to improve WSNs. Therefore, OFM-HNA enables access to available resources in the nodes through implementing a standard abstraction system that does not require access to the device. The OFM-HNA approach provides flexibility, adaptability, and reliability with control of the WSN by using models. These models deploy, manage, and update the network in the device, gateway, and enterprise levels. However, the proposed architecture does not provide any collaborative results of OFM functions with WSNs.

The Rescue and Crime Information in the Cloud (RCIC) [57] is based on SOM architecture. RCIC consists of a set of heterogeneous sensor nodes that form a cloud-based system in MANET [57]. The sensors send data to the cloud to process and analyze it. Then, the data is normalized through the middleware and transmitted to the Rescue and Crime Information System (RCIS) [57]. RCIS is a method that individually assesses secure data versus at-risk data. RCIS detects natural disasters or criminal activities. It can easily monitor any event by providing a fast response time. The simulation result of 500 sensor nodes shows that the power consumption and range size of each node is reduced by using clusters. Each cluster consists of 100 nodes executed in parallel. RCIC’s limitation is in its accuracy. It is not accurate enough to handle complex services or networks. The network uses a lot of data that causes processing delays. Even though the RCIS acts as a filter, it should enhance the database to filter unnecessary data. If this filtering takes place, overhead and processing delay of data will decrease and the network accuracy will increase.

Another SOM architecture called Service Mid-Tier Component (SMC) based on SOA is introduced in [58]. In this technique, each component is represented as a service within the middleware framework. This approach has a repository that includes various types of interfaces and a middleware. It handles any type of delivered request and then identifies a suitable interface from the repository and links it to the service. It can decrease overhead, storage space, and power consumption on each node in the network. Each layer should be independent of others because individual layers provide a self-contained module increase flexibility and scalability within the system, and protect individual data. In this case, the repository should use secure algorithms to establish interactions with the nodes. In [58], the proposed method is used to handle the traffic route between the sources and destinations; however, it should be optimized to increase quality of service in the system. In this approach, the authors need to evaluate additional applications in order to compare their results with other techniques.

Another middleware proposed is based on SOA through a web service [59]. It addresses different issues such as the serviceability of WSNs and the power efficiency for sensor application services [59]. The solution for serviceability occurs in the application of a Web Service Resource Framework (WSRF) within an Open Grid Service Architecture (OGSA) [59]. The power efficiency is solved by WSR. A web service based on the Markov Decision Process (MDP) produces query optimization techniques [59]. However, WSRF does not provide any quality of service for Service-Oriented for WSN applications [59], which is a critical issue especially in the case of massive data. The parameters of the quality of service such as data and process accuracy as well as the speed and failure rate of the operation should be considered. Data and system security are not addressed in this approach, and therefore can impact the system’s applications. Under OGSA, the WSRF transfers massive data between WSN applications; it should provide a method to control any loss or delay of data.

Similar to the preceding studies, the authors attempted to apply the quality of service (QoS). The active QoS Infrastructure of WSNs within SOM architecture is labeled as (QISM). The QISM was introduced in [60]. QISM is a software layer located between the protocol stack and applications [60]. It communicates with the layers by using API standards. The design of QISM has mechanisms and metrics that guarantee QoS for the entire network. The lifetime of the network and its application is increased through applied switching between the nodes [60]. By using two different regions of two different nodes, the network adjusts itself to the node with the highest power. The limitation of this approach is that there is no strategy for low-cost QoS monitoring processes, detection of QoS degradation, and data or service aggregation exists. The QoS degradation can be addressed by using the monitoring frequency approach [60]. This approach is more cost-effective than static or dynamic approaches. The management of the system and service should focus on the node and service level. The data aggregation in a sensor network can deal with simple data; however, it cannot deal with complex data.

Furthermore, many approaches of SOM architectures attempt to implement a flexible and scalable architecture with less cost. In this study, authors present an elastic sensor actor network (ESANET) environment [61], which proved to be more cost-effective. These applications run on top of SANET shared resources. ESANET is a software system that can bridge the gap between existing software and the next generation of SANET. The Role Oriented Adaptive Architecture (ROAA) is used to build a collaborative and adaptive ESANET software. The middleware architecture is used to achieve the goal of ESANET. The security mechanism is applied to the Nano kernel Middleware, an outside and inside security mechanism within the system. The limitation of this approach is that it does not provide details about the system’s performance, accuracy, and overhead.

The issues of integrating SOM architecture with sensor networks in the internet of things (IoT) technology were addressed in [62]. The authors proposed this type of SOA based on the middleware architecture. The features of SOA include a publish/subscribe mechanism that mediates communication between the IoT technology and the applications of existing automation systems. The publish/subscribe mechanism monitors traffic and manages asynchronous events. The IoT appears as either wireless sensors or identification tags. The middleware allows a smooth integration between heterogeneous technologies within applications [62].

According to [63], the existing Laboratory Information Management System (LIMS) at the Center for Life Science Automation (CELISCA) laboratories combined SOA with WSNs (SOA-WSNs) [63]. This approach relied on Sensor Web Enablement (SWE) and Sensor Observation Services (SOS) that provided the sensor measurement of data in different WSNs [63]. The architecture used a DPWS-based web service to assist in the cooperation, abstraction, and device orchestration of the LIMS services. In Life Science Automation (LSA), Carbon Monoxide (CO) and Hydrogen (H2) must be regulated by sensors [63]. Unfortunately, WSNs do not support these dangerous gases. However, SOA-WSNs in LIMS were designed to detect any of these risks and block any disasters within LSA to guarantee a valid analysis procedure. The LSA observation service analyzes the actual sensor readings and will release the necessary responses in the case of any abnormalities. The flexibility, usability, and extensibility of this architecture is increased through a developed WSN-based service infrastructure. In [63], the researchers claim that this approach decreases cost and setup times. However, since no results were provided, this approach cannot be fairly evaluated.

## 7. Service-Oriented Architectures Approaches for WSNs

This section discusses the latest approaches based on SOA. SOAs do not apply middleware architecture on their schema.

### 7.1. Network Discovery and Selection

Wireless mobile networks have a limitation due to the heterogeneous network environments [15]. The mechanism to discover and select the best network can be reduced during the transmission of network services that takes place when heterogeneous networks exist [15]. The Access Network Discovery and Selection Function (ANDSF) was proposed but still has challenges such as collecting and enabling network data from access networks, making available this information to be available for network discovery and selection, and updating this information in real time. The SOA provides a flexible mechanism to discover and select a network in wireless mobile networks [15]. The SOA is applied to ANDSF to process heterogeneous wireless mobile networking. Costs are reduced because the notification message consists of only an updated network state and does not contain the entire service description. Network service descriptions keep the most recently updated information at the network service registry. This mechanism helps discover and select the most optimal access network in real-time instead of republishing all network service descriptions. The system increases the capability of the network service description by using the capability matrix [15].

### 7.2. Healthcare Approaches

The Service Layers Over Light Physical Device (SYLPH) architecture [64] consists of layers added over the application layer in each WSN stack [64]. SYLPH is a unique architecture that helps in integrating SOA with WSNs that can be used to build a system based on Ambient Intelligence (AI) for maintaining patient information, which was presented in [64]. The AI provides an intelligent distributed system, allowing effective communication irrespective of location and time [64]. The SYLPH gateway is connected to different sensor networks by using various hardware interfaces. It enables two device types (either the same or different) to work together, such as ZigBee and Bluetooth devices. The system improves the healthcare monitoring of home-bound patients through a prototype system. The drawback of SYPLH is that it has not been tested in real-time.

Similarly, in [65], a unique framework based on SOA with Wireless Body Sensor Networks (WBSNs) and Web Services (WB) was proposed. The framework provides healthcare services to monitor elderly people and allow doctors and nurses to access patient information. This framework provides a mechanism to keep the healthcare data secure and private, based on the authentication mechanism which decides to allow or reject the user access request. This service helps elderly individuals by carrying a very lightweight and efficient biosensor. The feature of this framework includes reduced memory space, interoperability of service, maintenance cost through storing strange data in a central server, a fast response time, increased privacy, and throughput. The limitations of this framework include overhead, due to its use of XML and SOAP.

The concept of SOA is used in tele-monitoring. SunShine is integrated with distributed WSNs and the internet to perform complex tasks [66]. SunShine is a web-based system that manages data after collecting it, by analyzing the sensing data to see if it’s normal or not. However, applying SOA enables the creation of a Web Management System (WMS) for SunShine, providing flexible and reusable architecture. It can easily extend the sensing region coverage in web-based software design and monitor patients at all the times. The authors do not provide any security method to keep the patients’ data secure at all times, especially communication between clients and their doctors. Patients’ information is not sent or updated securely.

Correspondingly, the architecture of a tele-monitoring system can remotely monitor patient data. It has the ability to support efficient retrieval of information and addresses the QoS for visualizing data. SOA-based data architecture for healthcare monitoring with assistance from an algorithm that uses Extract Transform and Load (ETL) and Oracle Business Intelligence Enterprise Edition (OBIEE) is introduced in [67]. The drawback of this architecture is that it does not support heterogeneous sensors.

### 7.3. Open Geospatial Consortium with Sensor Web Enablement (OGC SWE)

Recently, internet services have applied Geographic Information Systems (GIS) that support environmental observations such as weather, a fire alarm, and indoor surveillance systems. As introduced in [68], a WSN Application Service Platform (WASP) is a novel sensor control service with web/GIS based architecture [68]. The WASP (acting as a cloud service) manages data through many data recovery points by sensors that are sent to the server for query by the user. The users are not able to identify between raw and processed data, which results in the loss of necessary information. The WASP is used to manage data and provides various mechanisms, such as data presentation, remote control functions, and security. The limitation of this approach is addressed in [69]; the sensor web enablement was developed to provide a solution for raw data identification and issues related to the mashup between WSN applications. The Sensor Web Enablement (SWE) is based on the Data Observation and Event Notification framework (SWEDOEN) [69] and has been used for smart home applications. This framework has a flexibility of application with WASP and can assign the action and message flows between SWE components. These approaches are not providing mechanisms for a WASP with GIS web service to handle large heterogeneous data in real-time. The middleware can handle a massive amount of this data by using different interfaces, languages, and content messages to convert data to fit the users’ needs. The accuracy and performance of their approach is not considered.

Moreover, OGC SWE s capable of real-time monitoring. The integration of WSNs into SOA by using a web service proxy linked to high-level SWE to low-level sensor platforms is presented in [70]. OGC SWE is applied for the sensor description, and observation with open Message Queue Telemetry Transport (MQTT) provides a suitable solution for low-level uplink from the WSN to the sensor web. The communication at the proxy layer is done through MQTT. The MQTT is used to solve the issue of one-way communication by using bidirectional communication for OGC SWE. This system is required for WSNs to have web-enabled remote management platforms, which allow data management API to manage and configure WSNs. The Sensor Planning Service (SPS) only describes the ideas but no real world tests were shown. The OGC SWE standard has challenges such as performance, robustness, and reliability. In [71], SOA provides Sensor Node Management Cloud (SeNoMa-cloud) software, which is extended on a proposed framework in [70]. SeNoMa is designed to manage the WSN configuration. The system deploys nodes in different locations of interest, for example, crop fields, and then assigns a sensor to the nodes, locates login, and transfers periods. The GeoSense system is used as a tool for clients to collect, analyze, and visualize the data. The system has many sensor nodes and base stations and can easily manage a WSN using SeNoMa-cloud by a virtual private network. The development of SeNoMa-cloud has to be suitable with OGC SWE. The OGC SWE has one-way communication in which it can only receive data/services from SeNoMa and send it to the cloud. This approach provides advantages for WSN management on multiple stations and deals with raw data. The sensor node management mechanism was designed to manage WSN configuration. This approach is limited because it increases overhead by using XML-based web service. An increase in the overhead could cause data transmission with low bandwidth. OGC SWE provides mechanisms to detect and determine failure, in order to reconfigure the system so that it can continue execution.

WSNs are widely used in many studies, such as agriculture control applications and natural resources. Different architectures are used in agriculture to provide an efficient platform for making decisions on how to manage crop planning. An Open Geospatial Consortium (OGC) with SWE that provides a direction for semantic standardization of sensor networks is presented in [72]. The components of SWE are SensorML (Sensor Model Language) and an SOS (Sensor Observation Service) [72]; it can be interoperable for processing data online [72]. The SensorML is XML and used to represent different features of a sensors’ system. It provides performance characteristics such as accuracy and the capability to describe the sensor system, process models, and connect sensor networks over the internet. The OGC SWE through SOA was implemented by using two distributed sensing systems.

### 7.4. WSN Cloud User Interaction

The new concept for WSN cloud is designed specifically to apply to a network as a service (NaaS), which provides solutions in large-scale WSNs for Service Orchestrating Architecture provisioning called (WSNs-SOrA). WSNs-SOrA enables WSNs to act as a cloud and is required to support SOA at all WSN tier infrastructure. The SOA enables another system to provide WSN infrastructure based on their needs, while allowing multi-systems to use the WSN. The service provisioning is done using XML [73]. This approach is one of the first state-of-the-art protocols proposing to combine WSNs with cloud computing [74]. In [75], methods that use sensor data by cloud users are presented. It designs service stacks, interfaces, and repositories based on SOA. The services allow communication between the cloud, WSNs, and the consumer. This architecture supports setup for WSNs which can collaborate, share data efficiently and easily determine the sensed data behavior. The issues of this WSNs setup is addressed through isolated sensor networks and non-collaborative approaches. The isolated sensor network drawbacks are solved by using one registry for sensor networks, and the challenges of non-collaborative approaches are addressed by designing a service stack. The heterogeneity issue is addressed by using SOA.

### 7.5. Configuration Service

The Service-Oriented system is used due to its capability to perform the service configuration in areas that have spatial and relevance constraints. This system has several mechanisms to improve the efficiency by configuring services and performing complex tasks in the input and output of data. The mechanisms of this system include reconfiguration and fault tolerance, and generic cost as well as centralized, distributed, and hybrid configuration modes. The generic cost function is used to integrate BaseCost and RelevancyCost. The system has the ability to detect any failure in service and reconfigure itself automatically [76].

### 7.6. Service-Oriented Device Architecture for Smart Environments

The Simple Object Access Protocol (SOAP) is deployed based web service on the node without a need to build it on the gateway. This approach supports and integrates into a legacy IT system by using SOA in a simple manner; this can support the heterogeneities at low level, without requiring additional middleware. The experiments of this architecture are done using Mulle, which is a resource-constraintsensor platforms. Every device consists of SOA interfaces, which can enable interaction with high-level business applications without using intermediate gateway protocols. An efficient lightweight TCP/IP stack combines with IwIP and gSOAP web service toolkit, increasing the processing time for SOAP messages. This design supports different network layers. The security is considered by using the DPWS, as the sensor nodes in this approach are behind a firewall enterprise. The approach is only suitable for noncritical applications. In this method [77], sensor data aggregation reduces transmission time and increases battery life is shown. The processing of SOAP messages generates overhead, but not as much as the message transmission. The limitation of this approach is the performance of overhead communication [77].

### 7.7. SOA Model for Sensor Networks

The Service-Oriented Model is designed for WSNs with internet (IP network) through different components such as Application Agent (AA), Resource Manager (RM), Register Agent (RA), and multi-gateways [78]. The architecture of these components performs as a service provider and the CQM (Complex Query Management) that exists among the WSN and internet from the gateway can be separated. This design provides a flexible architecture by using multi-gateways with RM. The architecture provides suitable mechanisms that guarantee all data from the sensors is transmitted correctly to the subscribed users. The system requires data to be located closest to the users and the filtering mechanism to be closest to the source. This mechanism should use a method to keep this data in a secure manner. The drawback of this approach is that it does not test in real-time [78].

### 7.8. Other Approaches

Recently, SOA has gained a lot of attention for providing flexibility in the designing of WSN applications. In [73], a method of service selection with flexible Service-Oriented Network Architecture (FSONA) addresses the issues of WSNs. These issues are increasing because of the lack of interoperability and the addition of new services or adaptation new protocols between the sensors and communication architecture. Addressing these issues provides a general communication between users, developers, and applications. In this architecture, a common platform connects the heterogeneous and homogeneous services [79].

Most of the existing routing protocol studies exploit SOA in WSNs. In [80], the path vacant ratio is used to find a group of disjointed paths from available ones and link them. The load balance and congestion control algorithms are used to check and control the load on multipath. The Threshold Sharing Algorithm (TSA) has the ability to divide each packet into many segments before transmitting it to the destination over the multipath based on path vacant ratio [80]. A secure and adaptive load-balancing multipath routing protocol based on AODV called Service-Oriented Multipath AODV [80]. The benefit of applying AODV protocol is to extend the load balance algorithm due to its routing protocol efficiency, without generating any congestion. SM-AODV provides secure data transmission and improves data confidentiality in Service-Oriented WSNs [80]. The features of multipath routing protocol include a secure transmission of data, independent applications, adaptive congestion control, and extensibility [80].

Another Service-Oriented approach supports QoS and real-time in Industrial Systems [81]. The SOA philosophies can be applied in the enterprise IT and the sensor network itself [81]. The enterprise IT system integrates into the sensor nodes by linking the Service Descriptions (SD). The linked data of the SD and RDF (Resource Description Format) addresses the problem generated through integrated enterprise IT system with sensor nodes [81]. The sensor motes interact with different service descriptions connected to other service descriptions by the Unified Service Description Language (USDL) method. The corresponding interfaces and the service description are located on/off the sensor or on both, which can lower cost reducing data on the sensor [82].

The flexible architecture is introduced in [83] for sensor networks based on web services and web mashup [83]. Web services build based on SOA. The data is provided through sensor nodes, and service is provided through WSNs for client applications and provided services, such as sensor nodes, to generate raw data. The raw data is processed and generated by different analyses, filters, complex processes, and web mashup, which provides value-added services. This architecture is adaptive SOA for designing WSNs. The services consist of the abstraction that can be used for developing WSNs applications. XML is used for representation and exchanging data between applications and the network. The WSN is integrated with the mashup, which is used to build different applications on top of the virtual ecosystem of services [83]. SOAP and HTTP modules manage communications. The SOAP should be presented in web mashup and sink nodes, with HTTP module in sensor nodes [83].

Additionally, SOA is applied in business applications. The SOA and mashup have allowed the enterprise to transfer complex applications through integrating the information over internal and external sources. It enables the user to take heterogeneous data from different sources. Therefore, it provides graphical tools called “enterprise mashup” for business users to select, integrate, and analyze data as needed. The approach addresses the collection of accurate and real-time information to satisfy business requirements based on enterprise location and the structure of the data [84].

Moreover, there are various concrete implementations of SOA approaches. A multi-SOA approach is designed to increase the efficiency and QoS of the system [36]. The WSN-SOA, a multi-level based on the existing SOA on the higher tiers with a protocol stack is presented in [36]. The SOA has the capability to handle the nodes with low capacity without generating an overhead of XML-based technology. WSN-SOA allows the SOA-based communication of low capacity sensors in the networks as MICAz motes. The multi-level via auto-configuration can enable all sensors to turn into reusable resources and allow the distributed collaboration between them. The “software stacks” help link between low capacity and full capacity nodes [36]. The extension of WSN-SOA stacks is introduced in [36]. It supports dynamic deployment of Service-Oriented cooperative tasks in the networks efficiently. The WSN-SOA is implemented on open source operating system TinyOS 2.1 (TinyOS Alliance) and develops WSN-SOA for Crossbow MICAz (Crossbow Technology, Inc., Milpitas, CA, USA) [37].

Similarly, the x-SOA approach [85] is related to previous approaches. There is X-SOA framework for sensor web service discovery mechanism, which is based on the Natural Language Query Processing (NLQP) by using semantic grammar [85]. The framework acts as the intermediate layer, called RPQ (Request Parser & Query generator), which supports interoperability between the service requester and the service registry [85]. A novel algorithm called Sensor Web Registry Services Discovery (SWRSD) is used in all steps of the processes of sensor service discovery [85]. The different layers can interact with each other by Unified Modeling Language (UML) sequence diagrams. The limitation of this architecture considers only the QoS function but does not deal with QoS non-functional. The non-functional is known to provide efficiency to the sensor web registry. In [86], the authors used the same mechanism and added QoS non-functional to the sensor web registry. Multi-layers of SOA framework are proposed for Sensor Web Service Discovery (SWSD) mechanisms that are based on the Natural Language Query Processing (NLQP) [86]. The architecture reduces the burden of novice requesters. The overhead decreases by converting user requests in XML or SOAP to other formats. The architecture has fewer capabilities for dealing with other QoS or for supporting different types of sensor web services. The limitation of this approach is that it tests only five sensor nodes and should be evaluated with additional sensors to obtain more QoS parameters. The power consumption, data aggregation, and delay should be considered with this approach.

The studies [87,88] proposed a generic framework approach based on web service which can be built as a standardized interface between external networks, applications, and WSNs. The implementation is based on Direct Service-Oriented Diffusion (DSOD) and the Service-Oriented Routing Protocol for WSN [87,88]. The SOA is implemented on the sensors. The security services are addressed in this architecture and provide Authentication, Authorization, and Accounting (AAA) mechanisms. The drawback of this approach is that accuracy is not considered. The name-centric service architecture framework based on the data/Content-Centric Network (CCN) for cyber physical system (CPS) can address the limitation provided by using transparent methods for accessing the services in the CPS. It implements a lightweight approach for WSNs which is called Content-Centric Networking Protocol for WSN (CCN-WSN) and can easily implement a gateway between CCN-WSNs and CCNx to build the SOA [89]. This approach still has limitations due to the named services required when coordinating naming in CPSs. This drawback should be addressed by using standard naming system for the CPSs.

The NanoSD is a service discovery protocol which designed for mobile, dynamic, and heterogeneous of WSNs [90]. The implementation of NanoSD provides a lightweight service discovery protocol for WSNs [90]. This implementation meets the requirements of service discovery, such as supporting mobility and dynamics in the network, running on heterogeneity nodes platforms, adapting to software modified/changed, and being flexible and easy to maintain. The heterogeneities of WSNs are supported in this architecture by providing a gateway library. The NanoSD protocol reduces packet size and communication overhead which can provide fast processing. The developer has the ability to select proper routing for WSNs and applications of the routing protocol [90].

The WSNs and SOA approaches are integrated for Intelligent Transportation Systems (ITS), which can obtain the best results for safety and security in its applications. This integration is useful to develop several ITS applications [12].

In addition, a WSN based on SOA with web service is used to detect collision, such as vehicles with motorway guardrails. The simulation applied to determine the propagation wave on guardrails uses the Finite Element Method (FEM) in real-time. This system improved the reliability of collision detections, reduced cost, and is easy to maintain [91]. This approach has packet collide limitation. Due to the receiver node being received, information from multi-sensors are transmitted at the same time.

## 8. Service Composition for WSNs

In this section we introduce an overview of Service-Oriented computing in sensor networks and ad hoc. Most approaches focus on SOM architectures and service composition still under research. In the next section, we discuss some approaches based on service composition for WSNs. The service composition is a design principle applied within the SOA, which is composing a massive service by combining many small services. The service composition is a method that combines and coordinates the aggregate of service and processes service entities into high-levels of application. For example, a controller service application requires the design service to control the other service. The service composition is responsible for allocating all required service to the service provider. The performance load balance, resource and end to end delay are studied well in service composition.

### 8.1. Service Composition with Persistent Queries (SCPQ)

The service composition can reduce the total number of solutions over the lifetime of persistent queries. Reduction in this number can decrease the total cost of service composition [92]. Routing in WSNs is used only to find a path from the source sensors to the receiver node. Thus, Service-Oriented query routing protocols are applied in order to guarantee a path from the source sensors to the sink and should also include service providers [92]. Two algorithms are applied to minimize energy consumption, which can provide service composition solutions for a persistent query. These algorithms are called Greedy and Dynamic Programming. The Greedy algorithm is applied to minimize the total number of service composition solutions during the lifetime of a persistent query. The Dynamic Programming algorithm uses the results of the Greedy algorithm to find a shorter path and reduce the total cost of service composition solutions. The time complexity of the Dynamic Programming algorithm is defined as O ((D/T) ^3) [92]. Similarly, another study uses the Greedy algorithm to select the best nodes. The middleware system service-based approach for WSNs provides QoS and context-awareness [93].

### 8.2. Service Centric Wireless Sensors Networks (SWSNs)

**Figure 11:**
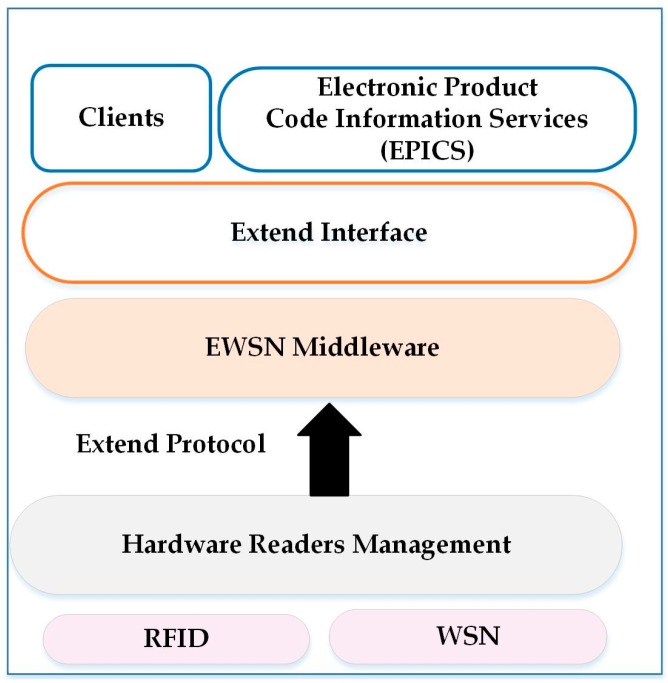
EWSN Sensor-based Architecture [94].

**Figure 12:**
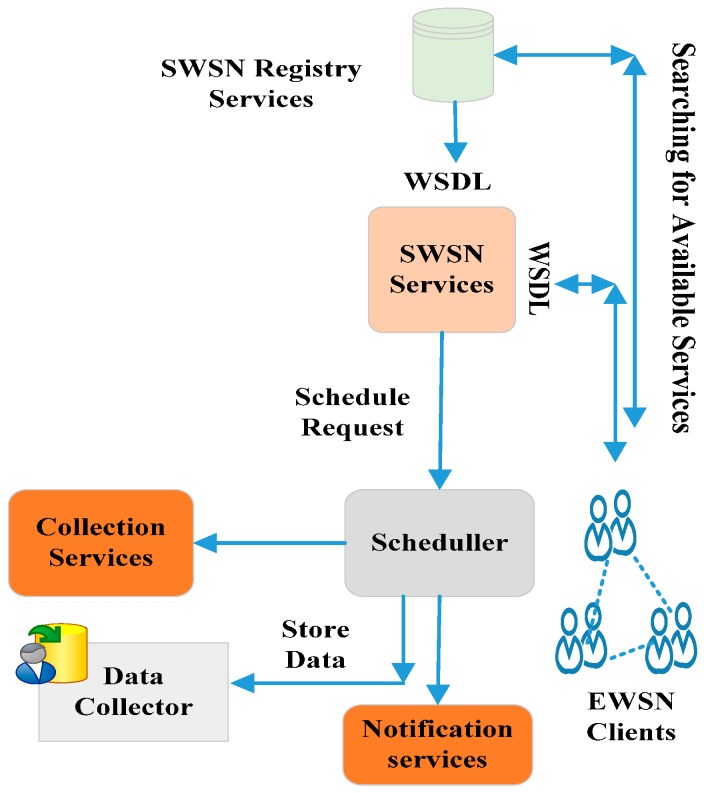
SWSN Dynamic Service Platform [94].

Flexible solutions are necessary to properly handle complex issues that arise within heterogeneity data and devices. SOA has the ability to control these types of data. The work presented in [94], the integration of the Extended WSNs and RFID tags within a web service, is called EWSN nodes. The framework is used to collect and share data from RFID and WSNs as shown in Figure 11. The studies propose the integration of EWSN schemes into the IoT as shown in Figure 12. The EWSN has challenges during the application phases in real-time. It cannot handle different operations and heterogeneities in the system or sensors and has difficultly executing the data. These challenges are addressed by applying SOA and EWSN to the service centric WSNs. This is referred to as intelligent SWSN nodes. Once a web service is applied to EWSN, any interoperability that existed between different applications, heterogeneities or dynamic systems is remedied. The Electronic Product Code (EPC) acts in the network as a mechanism that can process the data of the WSN and RFID. The EPC with SOA provides an easy way to integrate WSNs with RFID tags for IoT applications without the above-mentioned issues.

## 9. Analysis

Most of the existing approaches on SOM architectures and SOA for various WSN applications are highlighted. The proposed approaches attempted to address most of the WSNs challenges and are classified in three types. First, the approaches that applied different middleware architecture to achieve well-designed architecture for WSNs. Second, approaches that attempted to implement SOA for WSN without applying the middleware into the design. Third, an overview of the management and the service composition of some approaches that have remained relatively unexplored are shown.

### 9.1. The Service-Oriented Middleware (SOM) Architectures for WSNs

In our best knowledge, numerous SOM architectures for WSNs have been specifically designed to address the complexity issues that are related to resources and optimization of the pervasive technology. These approaches were aimed towards tackling the open issues that were previously identified in WSNs. None of the reviewed state-of-the-art approaches fulfil every requirement of the WSNs, as shown in Table 2, Table 3 and Table 4. The heterogeneities between sensor hardware and communication devices in large-scale WSN applications have difficulty executing data from different networks. The data/service aggregation aims to minimize energy consumption and network load on the sensor networks by optimizing the transmission data based on time and battery life. Some approaches do not provide any mechanisms that are independent of the middleware; instead, they depend on particular operating systems. The ESOA framework is built on LiteOS while MiSense is built over TinyOS. The support for heterogeneous multi-service composition highlights the enhancement of service interworking and provisioning to end-users, enabling service orchestration, and discovery at the middleware level. However, these mechanisms are only provided in USEME, OASIS, and ESOA approaches. On the other hand, the security mechanisms have been taken into account through different SOM architectures approaches like SOMM, ESOA, and SAWM. Data or service aggregation is supported in approaches like OASiS, MiSense, SensorsMW, and ESOA. However, most of these approaches do not provide specific implementation and mechanism details. In Table 2, a summary of Service-Oriented Middleware architectures are presented. These approaches are regarding the open issues in wireless sensor networks that identified previously. Table 3 highlights the representative SOM architectures for WSNs with the evaluation of its advantages and disadvantages. The implementation of these approaches offers relative limitations and strengths. Finally, the requirements and benefits of using SOM for WSNs are shown in Table 4.

### 9.2. Service-Oriented Architectures for WSNs

The SOA comprises of diverse notions, concepts, and technologies from a wide range of studies. Table 5, Table 6 and Table 7 show the comparative analysis of service-oriented architectures for WSNs. In this paper, state-of-the-art approaches based on SOA design for WSN are presented. Even though most well-known examples of SOA are web services, it is important to know that it is not limited to it. The biggest issue of applied traditional SOA into WSNs is that those are built on different platforms/operating systems (OS) without the use of middleware. The approach is considered to support general core functionalities independent of the platform and OS. None of these approaches supported the multi-service composition except for the FSONA approach. Table 5 shows the approaches that applied traditional SOA into WSNs that do not support middleware architectures. Some of these approaches provide general architecture with some limitations as shown in Table 6. In Table 7, the requirements and benefits of traditional SOA for WSNs. 

Security challenges and performance of data aggregation are not supported in most of approaches while only SODA and SYLPH approaches support security at a low level. In conclusion of this analysis, it is fair to comment that none of the reviewed approaches accomplishes all the requirements globally. The scalability, security, QoS, data aggregation, integration, and overhead limitations should be taken into account during the implementation processes of future designs.

### 9.3. Service Composition Architectures for WSNs 

Open issues of service composition shows that the adaptive service composition is required to have flexible composition methods that can enhance the scalability when the services are integrated into the network based on their availability. The SCPQ provides QoS requirements and decreases cost and power consumption. On the other hand, SWSN is capable of collecting information and reusing resources. The SCPQ approach does not address service composition languages on its design. In case of adaptive service composition, SWSN is based on web services. Meanwhile, SCPQ focuses on specific methodology such as service composition solution that is provided through the greedy optimal algorithm. However, SCPQ does not address service integration with the IoT, while the SWSN addresses this issue through web service. Table 8 shows the analysis of service composition architectures for WSNs.

In conclusion of the conducted analyses, Table 2, Table 3, Table 4, Table 5, Table 6, Table 7 and Table 8 represent SOM architectures, SOA, and services composition approaches with their requirements and evaluation of their advantages and disadvantages. The implementation of these approaches offers relative limitations and strengths. These approaches are reinforced through the abstraction level, sensors platform, extensibility, and reconfiguration. In this paper, the disadvantages of implementing a comprehensive framework and its limitations are considered. The main limitations that must be addressed are the heterogeneity of sensors networks, end-to-end security from the sensor to end users, QoS (solved through scalability and privacy), response time, and throughput. The service discovery mechanism should be available to assure the continuity of the service. It has the ability to discover any failures and replace them with the best available service during runtime. Since our framework deals with massive data, the communication efficiency should be increased with minimum cost, minimum overhead, and minimum energy consumption. The extensibility that can facilitate the inclusion of new networks or delete them without re-implementing the entire architecture should be taken into account.

## 10. Discussion

A number of research studies attempted to achieve the role of Service-Oriented software designs for network embedded system, but they only considered the software engineering aspect of it. The underlying computational platforms, such as SANET, and their limitations have not been considered. For security, none of the proposed approaches provide a comprehensive framework for different services or data secure architecture. The main issues with those approaches relate to the lack of consideration for accuracy in the architecture and data/service aggregation.

The middleware addresses the methods of publish/subscribe, virtual machine, database, and modular/macro programming. However, these solutions provide limited flexibility and interoperability based on interaction between end-users and high-level applications (clients).

Most SOM architectures for WSNs are based on heterogeneous services. These services impact the response time and network efficiency. There are different mechanisms and protocols to improve the efficiency of the services as well as the response time. SOM architecture deals with massive amounts of messages and events from various services that share those messages and events between the components of the system. In this case, the system must have the reliability to guarantee that the messages run correctly. The event management technique is used to increase reliability and QoS in WSNs. The QoS has the capability to decrease faults in communication as well as congestion. The QoS mechanisms can be selected from the best available network according to the QoS requirements and contract negotiations based on SLA [36].

There are several SOA protocols used in various architecture such as SOAP, WSDL, and DPWS. These protocols have addressed many challenges such as performance, overhead, exchange data, and security. DPWS used XML for data representation which represents slight limitation on the performance. And increase overhead [95]. The web service has two types of protocol [96]: Simple Object Access Protocol (SOAP) and Representational State Transfer (REST). The REST is an architectural-style application that can access resources/data. The SOAP is an XML-based message protocol which can wrap the business logic. The REST is better throughout and its response time is faster than SOAP. SOAP is used for message communication over SOA [85]. The description and discovery services are a web service description language (WSDL) and universal description discovery and integration (UDDI) [85]. These protocols are based on XML to share data between various computing systems. In order to keep the overhead low, these services use HTTP instead of SOAP for its implementation. In addition, DPWS-based web service is used in the architecture for the cooperation, abstraction, and device orchestration of services. In [97], DPWS uses different web service protocols to enable data exchange between data centric WSNs and other IP networks [97]. This approach uses a Service-Oriented Framework based on the DPWS gateway, which easily provides interconnection between IP networks and data centric WSNs and supports load balance and fault tolerance by using many gateway nodes for one WSN [97].

DPWS is based on middleware that can easily increase the overhead due to power consumption and latency [65]. Furthermore, it provides a secure service process through authorized parties, message integrity, and confidentiality. The DPWS is suitable for devices from certain regions. The DPWS cannot handle the overhead generated through web service, hence an efficient SOA implementation is used. Due to the overhead of SOAP and HTTP protocols, DPWS can be used. DPWS has the capability to secure services, since most of the applications do not require confidentiality for sensor data [65].

Most of the studies have not considered security mechanisms for sending the services/data from providers to the client, which can provide limitations to their approaches. In [33], a unique middleware based on Service-Oriented and message driven architecture for ambient aware sensor networks is presented. This approach does not provide a secure mechanism. Each node in the network should be registered to the main station to ensure security between sensor nodes and their station. The sensor nodes should encrypt their data through secure algorithms before sending it to their neighbors or the main station. Algorithms are needed to avoid any overhead or delay during processing and transmission of data. The QoS should also be taken into account to obtain more accuracy and a faster speed of operations.

In [56], SunShine is integrated with distributed WSNs in the internet to perform a complex task. However, this approach has limitations in sending and updating patient information in a secure manner. The authors do not provide any security method to keep patients’ data secure, especially during the communication between clients and their doctors.

**Figure 13:**
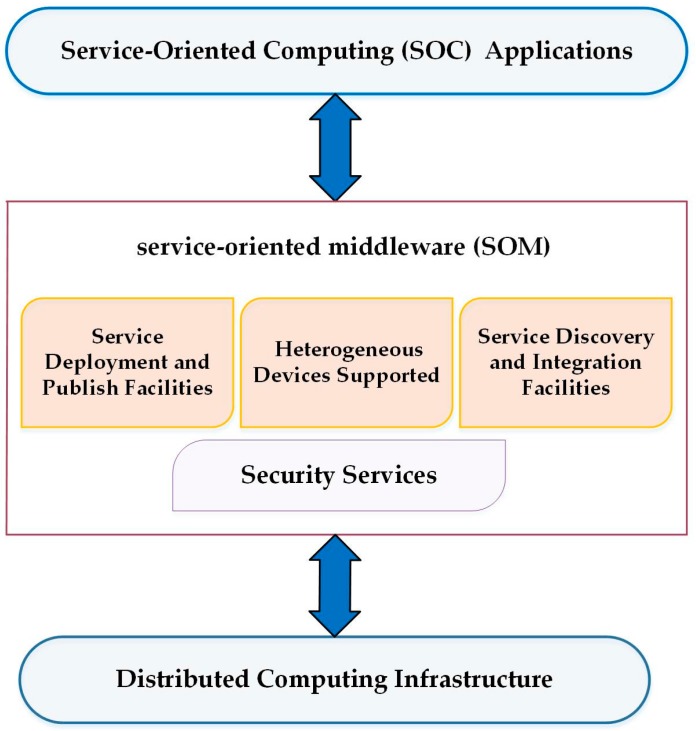
Generic Security SOM Architecture Framework [26].

In [98], a novel security mechanism is proposed for WSNs through SOA. In this architecture, the security measurement is used to address the flow of WSNs in a secure manner. The security is applied in the message level of the node, which is located near the cluster head and has the capability to recognize the identity of the sensor through SOA. The main goal of this approach is to reduce power usage and maximize the network’s lifetime by decreasing the size of processed information in the sensor nodes [98]. This method has the capabilities to interact, manage, and extend the system easily. The main problem with this approach is that the security is applied only at the message level, not the entire system. Each node should apply an encryption mechanism/algorithm to ensure that all data is generated in a secure manner. The applied algorithm should not impact or increase cost, overhead, or power consumption. The studies in [99] and [24] consider SOM architecture security requirements through a proposed generic framework that handles different security services independently as shown in Figure 13. These services support various security functionalities such as secure communications, messages protection, management trust, and access control.

The SOM architectures for WSNs should provide different functionalities that support the system. However, most of the studies on SOM architectures approaches do not provide all functionalities, including:

1. Secure executions and communications.
2. Deployment of service providers and advertisement.
3. Service consumer support to help discover/determine and register these services.
4. Support for QoS requirements.
5. Support for massive data and high level of communication load efficiently.
6. The ability to view the heterogeneities of the underlying WSNs, which are hidden by abstractions.
7. The ability to interoperate with multi-devices and systems.
8. Client application service transparency.
9. The ability to auto-modify and auto-discover mechanisms.
10. Configurable services.

Therefore, SOM architectures approaches for WSNs are based on heterogeneous services or devices; the efficiency of these services is impacted due to the response time and network lifetime. The response time of these services should be improved to increase their efficiency through UDP-based SOAP without the need for HTTP [100].

SOM architecture deals with massive data, messages and event notifications that are generated from different services and shared between different components [100]. In this case, the system reliability should ensure that these messages are delivered on time and are reliable. The reliability and QoS in WSNs are achieved by using event management mechanisms. However, some issues can be addressed by using QoS mechanisms such as congestion and faults communications, which are introduced in the OASIS and SensorsMW approaches. These approaches are developed by through selecting the most suitable available network based on QoS and service level agreements. The middleware has the ability to separate the application logic from the system logic.

## 11. Conclusions

The representative SOM architectures, SOA, and the services composition approaches with their requirements and evaluation of their advantages and disadvantages are presented in detail. The implementation of these approaches offers relative limitations and strengths. These approaches are reinforced through the abstraction level, sensors platform, extensibility, and reconfiguration. The main contribution of this paper is design, implementation, and validation of SOM architecture for various applications and environments based on WSN technologies. These requirements enable discovery, improved access, and sharing of the network service and data resources. Moreover, complex services can be achieved through an efficient execution of internetworking services and heterogeneous networks. These features allow the development of sensors based on the services of a third-party network. The analysis of the state-of-the-art SOM architectures foundations in sensor networks shows that most of the issues and challenges, not addressed in published approaches, have been discussed. Therefore, these architectures are designed to consider and address complexities related to the resources of the sensor networks. Most existing SOA and WSN-based middleware architectures do not address heterogeneous challenges. The main limitations that must be addressed are the heterogeneity of sensors networks, end-to-end security from the sensor to end users, QoS (solved through scalability and privacy), response time, and throughput. The service discovery mechanism should be available to assure the continuity of the service by discovering any failures and replacing them with the best available service during runtime.

## 12. Recommendations for Future Work

The motivation of this literature review is to contribute to research on the distribution of SOM architectures and implementation of a comprehensive SOM architectures framework for WSNs. To accomplish this, there are emerging approaches for example the SOM architecture to address the heterogeneity of the data that comes from different sensors. In the future work, along with the SOM architecture, Machine Learning (ML) must also be used as part of the services which facilitates the classification of heterogeneous sensors. Our proposal work adopts SOM architecture platform and implements a pervasive in-network service approach. This contribution addresses multi-service composition that can minimize the overhead in data transmission and data processing by using JSON standard format. Since our framework deals with massive data, the communication efficiency will be increased with minimum cost, minimum overhead, and minimum energy consumption. The extensibility that can facilitate the inclusion or exclusion (depending on the requirements) of new networks without re-implementing the entire architecture will be considered.

## Figures and Tables

**Table 1:** A Comparison of Different Middleware Architectures Approaches.

Middleware Approaches	Scalability	Heterogeneity	Ese to Used	Power Awareness	Application Type	Security	QoS
Database Approach	Not Supported	none	Yes	None	Event driven applications	None	None
Virtual Machine Approach	Supported	Not fully Supported	Little	Supported	Dynamic Applications	Yes	None
Message Oriented Approach	Supported	Not fully Supported	Yes	Supported	Event driven applications	Little	None
Modular Approach	Supported	None	Yes	Supported	Dynamic Applications	Yes	None
Application Driven Approach	Supported	None	Yes	None/Partial	Real-time applications	None	Yes

**Table 2:** Comparative Analysis of Service-Oriented Middleware Architectures for WSNs.

**SOM Architecture**	**Platform Type**	**Operating System/Platform Independence**	**Software Applications and Communication Model**	**Data/Service Aggregation**	**Heterogeneity**
USEME [45,46]	WSANs	Independent with in-network middleware	Abstract programming language (APL)	Not Supported	Not Supported
OASIS [34,35]	WSNs	Independent with in-network middleware (middleware is implemented on Mica2 mote hardware Platform)	Application development based on the separation of concerns (SoC)	Supported	Supported
MiSense [42]	WSNs	Built on top of TinyOS operating system	Programming Interface and Services Extensions	Supported	Not Supported
SOMDM [47]	WSNs	Independent with in-network middleware	Implemented based on Ambient Programming Model with the ported code in GALS by using Tiny GALS given by TinyOS	Not Supported	Not Supported
TinySOA [53]	WSNs	Independent with in-network middleware	Not Supported	Not Supported	Not Supported
SensorsMW [49]	WSNs	Independent with in-network middleware	Not Supported	Supported	Not Available
SAWM [52]	WSNs	Middleware for WMSNs	Infra-red cameras are applied to decrease the power consumption	Not Supported	Supported
Mob-WS [48]	WSN	Independent with in-network middleware	XML for the messages instead of using any transport protocols	Not Supported	Not Available
SOMM [51]	Distributed Enterprise systems	Independence with in-network middleware	Programming tasks based on byte-code	Not Supported	Supported
ESOA [54]	WSN	Built on top of LiteOS operating system	Not Supported	Supported	Supported
**SOM Architecture**		**Multi-Service Composition**	**Services**		
USEME [45,46]		Supported	Configuration Publication and Discovery [45,46] Command and Event Invocation and Communication [45,46] Real-Time Constraints [45,46] Group and Event Management Routing Protocol		
OASIS [34,35]		Supported	Node Manager [34,35] Service Discovery Protocol and Composer [34,35] Object Manager [34,35] GALSC queue ports [34,35]		
MiSense [42]		Not Supported	Event detection Data aggregation Topology management Routing		
SOMDM [47]		Not Supported	Not Available		
TinySOA [53]		Not Supported	Discovery Sensor Reading Internal Services Client Network Registry Events Registry External Services Server		
SensorsMW [49]		Not Supported	Data measurement Network maintenance Event notification		
SAWM [52]		Not Supported	Not Available		
Mob-WS [48]		Not Supported	Not Available		
SOMM [51]		Not Supported	service registry several servers		
ESOA [54]		Supported	Coordination and Service Discovery Performance, Monitoring and QoS		

**Table 3:** Advantages and Disadvantages of SOM Architectures for WSNs.

SOM Architecture	The Features and Advantages	Disadvantages
USEME [45,46]	Deals with the changes in the web service (WS) Supports a set of real-time management constraints Allows the programmers to use the programming task of the wireless sensor and actors network (WSAN) easily	Not considered security and hardware resources management Not support any mechanism to handle a large of data and high communication loads efficiently Not supports interoperability with various systems and devices Not provides a secure communication/execution Cannot integrates with other systems Not supports interoperability with various systems and devices
OASIS [34,35]	Development of environment based on separation of concerns Supports the node management QoS Dynamic service discovery Failure detection	Not provides a secure communication/execution Cannot integrates with other systems Not supports self-organization mechanisms Not supports interoperability with various systems and devices
MiSense [42]	Content based publish/subscribe service Provide programming API Supports data management	Not support configurable services Not supports self-organization Not provides a secure communication/execution Not support QoS Increase power consumption and processing time
SOMDM [47]	Decreased the data processing load by using multi-component architecture Supports DBMS Notification and data filtering techniques Handle a large of data and high communication loads efficiently	Not support configurable services Not supports self-organization Not provides a secure communication/execution Not support QoS
TinySOA [53]	It provides web service for internet Apps to access WSN Supports multiple programming language	Not support configurable services Not supports self-organization Not provides a secure communication/execution Not support QoS
SensorsMW [49]	The QoS configuration is provided by service level Providing mechanism for the application to manage WSNs	Not supports self-organization Not provides a secure communication/execution Not support nodes with low capacity
Mob-WS [48]	Increases the scalability	Not provides a secure communication/execution Not support QoS
SOMM [51]	Supports multimedia transmission Ability to reduce the cost of development applications Supports scalability and Supports network level heterogeneity	Overhead Not support any mechanism to handle a large of data and high communication loads efficiently Not very easy to use due to its implementation that used a comprises byte code
SAWM [52]	Provides secure architecture and modifiable	Not provides a secure communication
ESOA [54]	Allows users to develop new applications through mix-and-match services without any programming efforts by developers Supports the heterogeneous of WSNs and execute various applications on multi-platforms It can integrate with other systems Provides a secure communication/execution through QoS composition	Not provides mechanism to handle a data collection of user to the services Not applied in real time

**Table 4:** The Requirements and Benefits of Using Middleware Architectures for WSNs.

SOM Architecture	The Requirements	The Purpose of Middleware Architecture
USEME [45,46]	The configurable service Auto discovery techniques of the service providers Middleware allows the application executing and running in the network in secure way and easier to update anytime Dealing with a large amount of data and increase communication load efficiently The consumer service supported to detect and use register service	Middleware provide general-services such as configuration, invocation, and communication managements
OASIS [34,35]	The heterogeneity of underlying environments is hidden by applying abstraction such as protocols and languages The consumer service supported to detect and use register service Runtime is supported for the service provider to deploy services Support QoS Dealing with large amount of data and increase the communication load efficiently	Minimize the resource requirements
MiSense [42]	The heterogeneity of underlying environments is hidden by applying abstraction such as protocols and languages The consumer service supported to detect and use register service Runtime is supported for the service provider to deploy services Dealing with a large amount of data and increase communication load efficiently Interoperability with different device or system has flexibility to access its services by the high level interface	Data Aggregation Events detection Resource and Topology management
SOMDM [47]	The heterogeneity of underlying environments is hidden by applying abstraction such as protocols and languages Interoperability with different device or system Dealing with a large amount of data and increase communication load efficiently low overhead data filter mechanism	Allow sensor to handle data from ambient aware sensor networks Reduce data processing loads by using multi-component architecture
TinySOA [53]	The heterogeneity of underlying environments is hidden by applying abstraction such as protocols and languages The consumer service supported to detect and use register service Can integrates with other system	Discovery data readings Actuators management, and network communications
SensorsMW [49]	The heterogeneity of underlying environments is hidden by applying abstraction such as protocols and languages Configurable services Can integrates with other system Dealing with a large amount of data and increase communication load efficiently Interoperability with different device or system Support requirement for QoS	Supports dynamic management of heterogeneous data Provides QoS configuration by service level
Mob-WS [48]	Used as back end resources to reduce the complex Asynchronous services	Provides the best management and representation of wireless networks
SOMM [51]	Support Multimedia Support QoS, Virtual machine (VM), Mobile Agents, and Tuple space provides highly scalable platform by using SOA Energy efficiency is increased for the application modification The Mobile Agents and Code Repositories are used to enable the Node to be reprogrammed Handle heterogeneous nodes with different capabilities	Provides Security Hardware resource management Supports QoS
SAWM [52]	The architecture is easy to update used less memory for processing the programming codes processed in real-time Provide low cost during the transmission decrease power consumption	Provides secure architecture
ESOA [54]	Support requirement for QoS Interoperability with different device or system	Coordination, Monitoring, Conformance, QoS and Service Discovery

**Table 5:** Comparative Analysis of Service-Oriented Architectures for WSNs.

SOA Approaches	Operating System/Platform Independence	Type of Software Applications	Multi-Service Composition
SODA for Smart Environment [71]	Mulle Sensor Platform (resource constrained sensor platform)	Built upon the gSOAP toolkit with TCP/IP stack-lwIP	Not Supported
SOA Model for Sensor Networks [72]	Not Supported	Built on different applications such as Agent Register, Resource Manager, and Multi-gateway	Not Supported
WSNs Cloud User Interaction [73,74,75]	SOrA uses different platforms as TelosB and SunSPOT and acts as Node Network Tier [73] Stack of Services, Interfaces and Repositories [74,75]	Done by XML	Not Supported
FSONA [79]	Not Supported	Developed with Java Platform	Supported
Healthcare Approaches	SYLPH [64] Wireless Body Sensor Networks (WBSNs) [65] SunShine [66]	Built on ambient intelligence (AI) [64] Java (JDK 1.6, Apache tomcat server 6.0.) [65] and Java EE5 platform of NetBeans [66]	Supported
OGC-SWE standards (Web Service)	**WASP** has two sides ZigBee enables nodes communicate hop by hop with each other Software service using HTTPS protocol [68,69] **SeNoMa-Cloud** [70,71] A MQTT broker, ActiveMQ Apollo **SensorML** [72]	Built smart home system uses the SWE standard	None
Configuration Service [76]	Middleware Framework	Evaluation in CORE and EMANE	Not Available

**Table 6:** Advantages and Disadvantages of SOA for WSNs.

SOA Approaches	The Features and Advantages	Disadvantages
SODA for smart environment [71]	Support the Security, and heterogeneities at low level Not required additional middleware transmission time is reduced and battery life is increased by using Sensor data aggregation	Performance overhead communication while processing of SOAP messages but not as much as messages transmission Performance measurement effect on latency SOAP-based web services are required parse verbose XML documents
SOA Model for Sensor Networks [72]	Provide an efficient architecture Secure communication protocol Efficiently collecting data from WSNs	Does not test in real time
WSNs Cloud User Interaction [73,74,75]	WSN-SOrA and SOA have solutions and the ability to support infrastructure reuse [73] Enable data sharing in efficiently [74,75]	Overhead
FSONA [79]	Process heterogeneous wireless mobile networking. Costs are reduced	Overhead
SYLPH [64] WBSNs [65] SunShine [66]	provides a flexible distribution of resources SYLPH and capable during performance time to add new component [64] Decreases memory space, interoperability of service, maintenance cost, fast response time, high privacy, and throughput. This technique was improved the QoS to make decision and time warning generation the authentication mechanism and lightweight and efficient biosensor [65] Collecting and managing then analyzing data [66] Cost reduces [66] It modify the requirement of monitoring [66]	SYPLH is that it has not been tested in real time [64] Framework has overhead due to the use of XML and SOAP in the system [65] Not support Security [66]
OGC-SWE standards (Web Service)	**WASP** It process the raw data from WSNs [68,70] **SeNoMa-cloud** [70,71] WSN and SeNoMa-Cloud Services communicate with each other by using MQTT broker and ActiveMQ Apollo Small packet handles by using MQTT protocol Deals with raw data [64,65] **SensorML** Provide Accuracy Ability to describe the sensor system	WASP Not provides mechanism of how WASP with GIS web service is handling large heterogeneous data in real time [68,70]. It provides mechanisms to detect and determine failure [70,71]. Overhead by using XML based web service [72].

**Table 7:** The Requirements and Benefits of Applied SOA for WSNs.

SOA Approach	The Requirements
SODA for Smart Environment [71]	Support the heterogeneity Performance measurement effect on latency. The overhead that is related to SOAP message process was small when compared to messages transmission
SOA Model for Sensor Networks [72]	Multi-gateway uses to solve the issue of congestion that generate by using one gateway Authentication user Data should be located near the users and filter data near to distention Ability to linked various protocols that can be used for WSN
WSNs Cloud User Interaction	NaaS requires the WSN supporting Service-Oriented software architecture Non-collaborative [74,75]
FSONA [79]	Interoperability between service Supports QoS and run time Integrated with other system Service abstraction and discovery
SYLPH [64]	The devices are not requiring any features as large memory to communicate with SYLPH Improves the system security and efficiency for care services
OGC-SWE standards (Web Service) [68,70]	SWE standard helps to discovery sensors data and the interoperability Supporting the data detection Data retrieval increase for WSN through remote control Provide user authorized SWE standard helps to discovery sensors data and the interoperability Supporting the data detection
ANDSF	Solved problem the overhead between access networks and the service registry Provide mechanism for updating network states information in real time and service description
Healthcare Approaches	Supports efficient information retrieval Achieve the desired QoS in WSNs Support the heterogeneous and asynchronous
Configuration Service [76]	Adaptation at Runtime Reduce cost

**Table 8:** Analysis of Service Composition Architectures for WSNs.

SOA Approaches	Service Composition Programming	Active Service Composition	Services Integrated with IoT	Advantages	Disadvantages
SCPQ [92,93]	Not Supported	Service based on Greedy algorithm	Not Supported	QoS and context-awareness Minimizes Cost and energy consumption	None
Intelligent SWSN Middleware [94]	Proprietary semantic annotations for WSDL and XML	Semantic Web Services	Interoperability using WS-specifications	Collects information through the nodes can be reusable resources in the real world	Data redundancy Network dynamics Energy balancing and Traffic congestion problem
